# Biofortified Crops Generated by Breeding, Agronomy, and Transgenic Approaches Are Improving Lives of Millions of People around the World

**DOI:** 10.3389/fnut.2018.00012

**Published:** 2018-02-14

**Authors:** Monika Garg, Natasha Sharma, Saloni Sharma, Payal Kapoor, Aman Kumar, Venkatesh Chunduri, Priya Arora

**Affiliations:** ^1^National Agri-Food Biotechnology Institute, Mohali, Punjab, India

**Keywords:** malnutrition, biofortification, transgenic, agronomic, breeding

## Abstract

Biofortification is an upcoming, promising, cost-effective, and sustainable technique of delivering micronutrients to a population that has limited access to diverse diets and other micronutrient interventions. Unfortunately, major food crops are poor sources of micronutrients required for normal human growth. The manuscript deals in all aspects of crop biofortification which includes—breeding, agronomy, and genetic modification. It tries to summarize all the biofortification research that has been conducted on different crops. Success stories of biofortification include lysine and tryptophan rich quality protein maize (World food prize 2000), Vitamin A rich orange sweet potato (World food prize 2016); generated by crop breeding, oleic acid, and stearidonic acid soybean enrichment; through genetic transformation and selenium, iodine, and zinc supplementation. The biofortified food crops, especially cereals, legumes, vegetables, and fruits, are providing sufficient levels of micronutrients to targeted populations. Although a greater emphasis is being laid on transgenic research, the success rate and acceptability of breeding is much higher. Besides the challenges biofortified crops hold a bright future to address the malnutrition challenge.

## Introduction

“Biofortification” or “biological fortification” refers to nutritionally enhanced food crops with increased bioavailability to the human population that are developed and grown using modern biotechnology techniques, conventional plant breeding, and agronomic practices. The United Nations Food and Agriculture Organization has estimated that around 792.5 million people across the world are malnourished, out of which 780 million people live in developing countries (1). Apart from this, around two billion people across the world suffer from another type of hunger known as “hidden hunger,” which is caused by an inadequate intake of essential micronutrients in the daily diet (2, 3) despite increased food crop production (4). Besides this overnutrition is growing matter of concern.

So far, our agricultural system has not been designed to promote human health; instead, it only focuses on increasing grain yield and crop productivity. This approach has resulted in a rapid rise in micronutrient deficiency in food grains, thereby increasing micronutrient malnutrition among consumers. Now agriculture is undergoing a shift from producing more quantity of food crops to producing nutrient-rich food crops in sufficient quantities. This will help in fighting “hidden hunger” or “micronutrient malnutrition” especially in poor and developing countries, where diets are dominated by micronutrient-poor staple food crops (5).

Traditionally, vitamins and minerals have been provided to the masses through nutrient supplementation programs, but it falls short of the goals set by the international health organizations as the supplementation programs rely on external funding that is not guaranteed to be available from year to year. Other limitations are purchasing power of poor people, their access to markets and health-care systems, and lack of awareness regarding the long-term health benefits of these nutrient supplements (6, 7). Hence, biofortification of different crop varieties offers a sustainable and long-term solution in providing micronutrients-rich crops to people. Furthermore, biofortified crops with increased bioavailable concentrations of essential micronutrients are deployed to consumers through traditional practices used by agriculture and food trade which therefore provides a feasible way of reaching undernourished and low income group families with limited access to diverse diets, supplements, and fortified foods. From an economic viewpoint, biofortification is a one-time investment and offers a cost-effective, long-term, and sustainable approach in fighting hidden hunger because once the biofortified crops are developed; there are no costs of buying the fortificants and adding them to the food supply during processing (8–14). Furthermore, in the next few decades, a major population increase might take place in the developing world and with the changing climatic conditions; achieving food security will pose a greater challenge (15, 16). Thus, organizations such as the World Health Organization and the Consultative Group on International Agricultural Research (CGIAR) have included the development of nutritionally enhanced high-yielding biofortified crops as one of their main goals (17).

## Necessity and Socioeconomic Development Derive Biofortification Research

**Figure 1:**
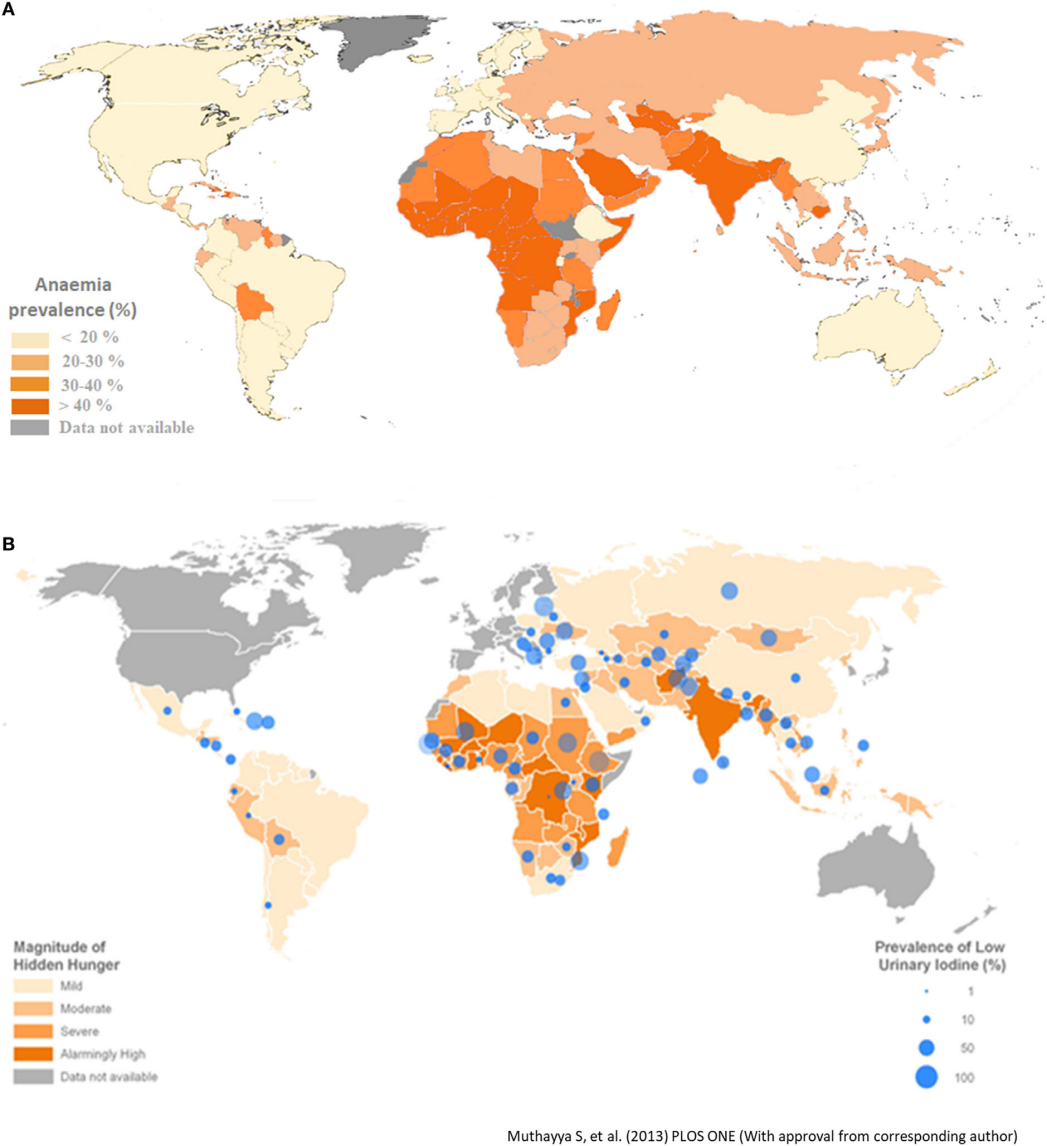
**(A)** Prevalence of anemia in different parts of the world. Developing countries in Africa and Asia have high prevalence of anemia [Data from Stevens et al. (30)]. **(B)** Global map representing hidden hunger index and low urinary iodine concentration (3).

Humans require around 40 known nutrients in adequate amounts to live healthy and productive lives (Table 1). The mineral elements—sodium, potassium, calcium, magnesium, phosphorous, chlorine, and sulfur—are classified as essential nutrients that are required in small amounts in the body. The other class of essential nutrients required in very small amounts in the human body are termed as micronutrients—namely iron, zinc, copper, manganese, iodine, selenium, molybdenum, cobalt, nickel, and vitamin A (18). Collectively, these nutrients play crucial roles in humans and dictate our physical and mental development (19). Many micronutrients act as cofactors for the functioning of various enzymes in the human body and thereby regulate important functions and metabolic processes in our body (20). For humans, agricultural products are the primary source of nutrients, especially for those living in developing countries (21–23). However, the diet of the population based on cereals such as rice, wheat, cassava, and maize contain insufficient amounts of several nutrients such as vitamin A, iron, zinc, calcium, manganese, copper, iodine, or selenium with respect to meeting daily requirements. These nutrient deficient agricultural products cannot support healthy lives and can result in poor health, sickness, increased morbidity and disability, impaired development, stunted mental and physical growth, diminished livelihoods, and reduced national socioeconomic development (24–29). Childhood stunting prevalent in many developing countries is associated with micronutrient malnutrition in children starting from fetal development to 4 years of age (25). Micronutrient deficiencies affect about 38% of pregnant women and 43% of pre-school children worldwide. More than 30% of the world’s population has been reported to be anemic (30) and suffering from hidden hunger. The prevalence of anemia is more in developing countries compared with developed countries. Africa and South-East Asian countries are most affected (Figures 1A,B). Estimates have indicated that approximately half of this is attributed to iron deficiency (31). Hence, micronutrient malnutrition is the major challenge in many developing countries. Another important point of consideration is uneven distribution of the nutrients among different plant parts (32). For example, the iron content is high in rice leaves, but low in polished rice grain. Apart from under nutrition, growing incidence of overnutrition leading to problems of overweight and in particular, high rate of diabetes is a matter of concern. Consequently, biofortification is also directed toward enhancing the contents of desired micronutrients in the edible portion of crop plants. Nutritional targets for biofortification include elevated mineral content, improved vitamin content, increased essential amino acid levels, better fatty acid composition, and heightened antioxidant levels in crops (12). Biofortification of crop plants can provide enough calories to meet the energy needs along with providing all the essential nutrients needed for sound health. Furthermore, biofortifying the crops which are consumed by the poor population of the world can significantly improve the amount of nutrients consumed by this target population (33).

**Table 1:** Essential micro- and macronutrients required for good human health.

Micronutrients		Macronutrients		
Micro-minerals	Vitamins	Amino acids (essential)	Fatty acids (essential)	Macro-minerals
Fe	A (Retinol)	Histidine	Linoleic acid	K
Zn	D (Calciferol)	Isoleucine	Linolenic acid	Ca
Cu	E (α-Tocopherol)	Leucine		Mg
Mn	K (Phylloquinone)	Lysine		S
I	C (Ascorbic acid)	Methionine		P
Se	B_1_ (Thiamin)	Phenylalanine		Na
Mo	B_2_ (Riboflavin)	Threonine		Cl
Co	B_3_ (Niacin)	Tryptophan		
Ni	B_5_ (Pantothenic acid)	Valine		
	B_6_ (Pyridoxine)			
	B_7_ (Biotin)			
	B_9_ (Folic acid, folacin)			
	B_2_ (Cobalamin)			

## Biofortification Pathway Includes Several Approaches

**Figure 2:**
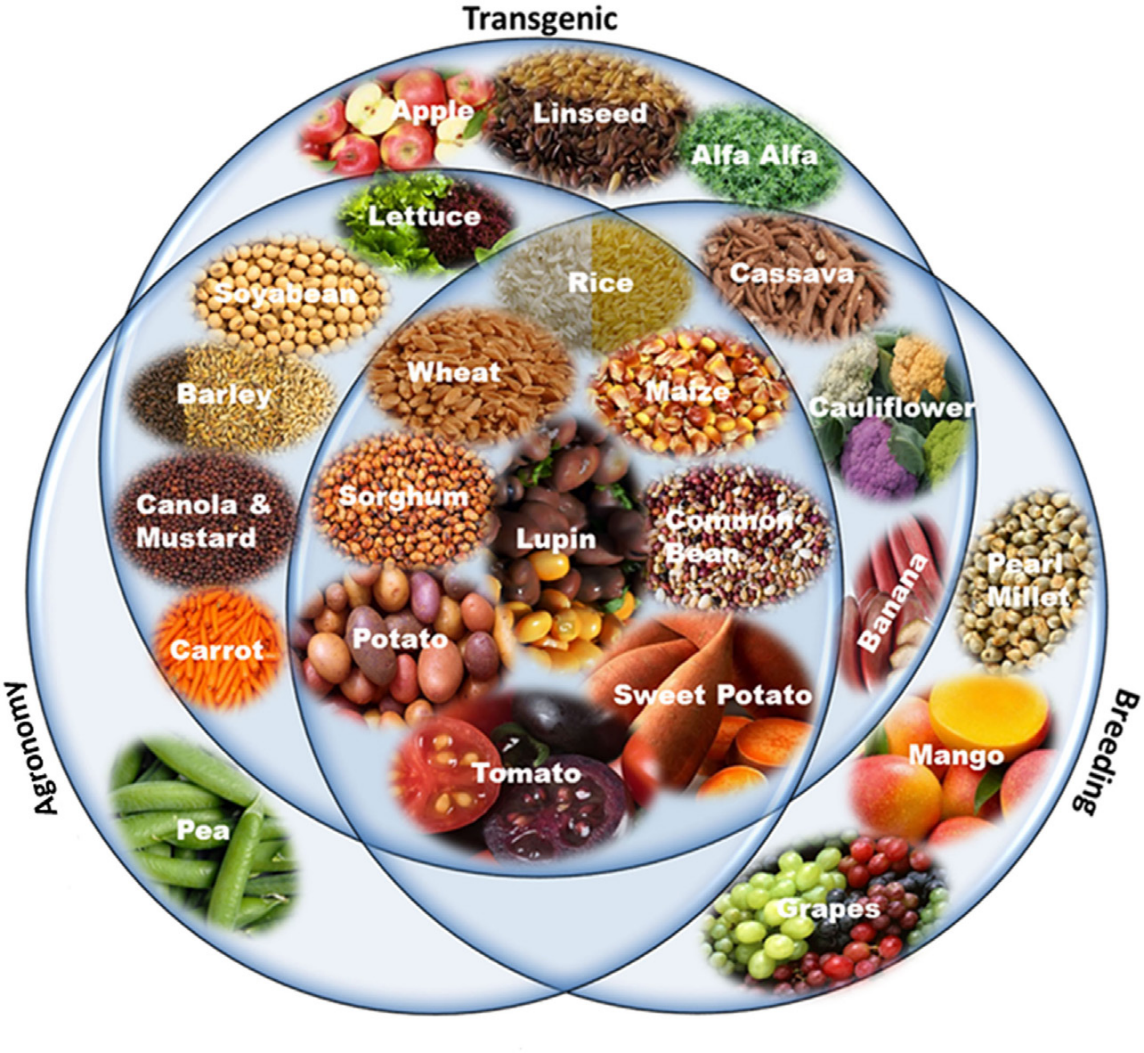
Biofortified crops generated by different approaches: transgenic, agronomic, and breeding. Staple cereals, most common vegetables, beans, and fruits have been targeted by all three approaches. Some crops have been targeted by only one or two approaches depending on its significance and prevalence in the daily human diet.

**Figure 3:**
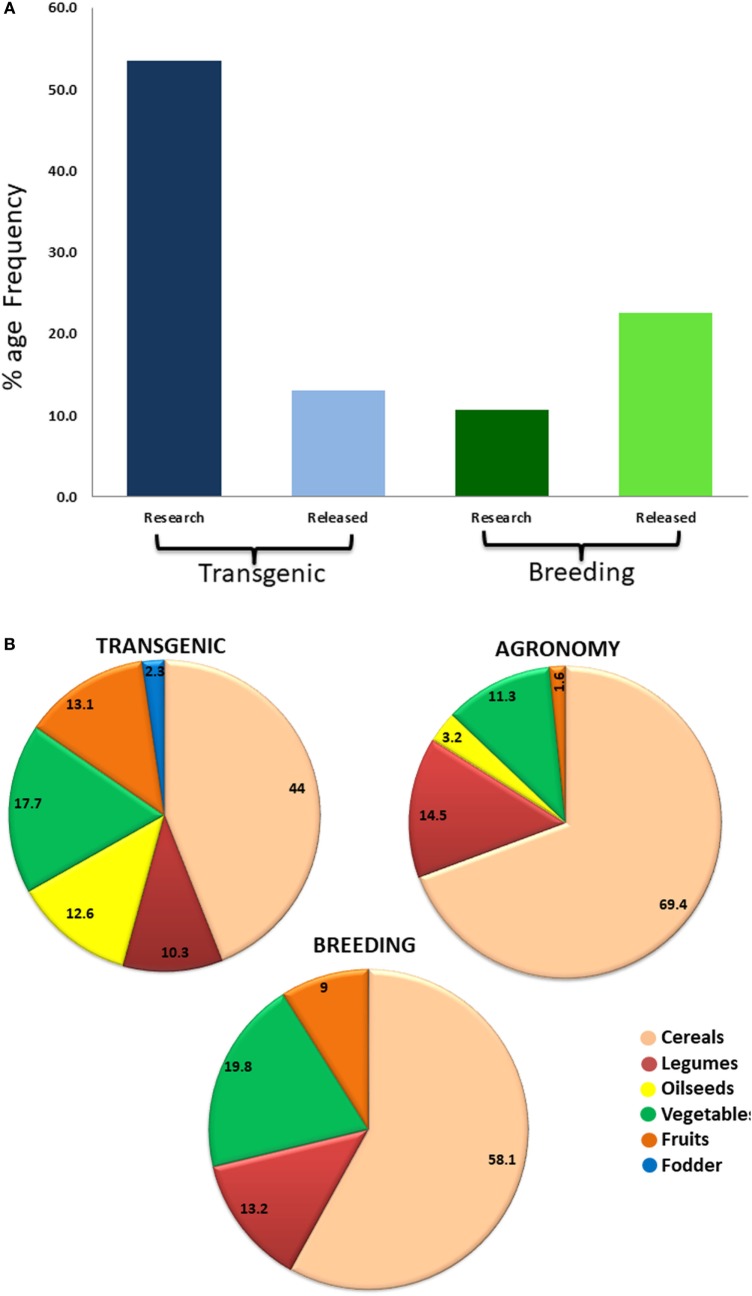
Representation of reported biofortified crops by transgenic, agronomic, and breeding means. **(A)** Comparison of transgenic and breeding approaches of biofortification in terms of relative research and release of commercial crops. While higher emphasis is being laid on transgenic-based biofortification, success rate in terms of cultivar release is higher for breeding-based approach. **(B)** Percentage of different crops biofortified by different approaches. Cereals have been biofortified in largest number by all three biofortification approaches. Legumes and vegetables have also been targeted by all the approaches in almost equal percentage. Transgenic approach covers highest number of crops. Oilseed crops have been mainly targeted by transgenic approaches due to limited genetic variability.

**Figure 4:**
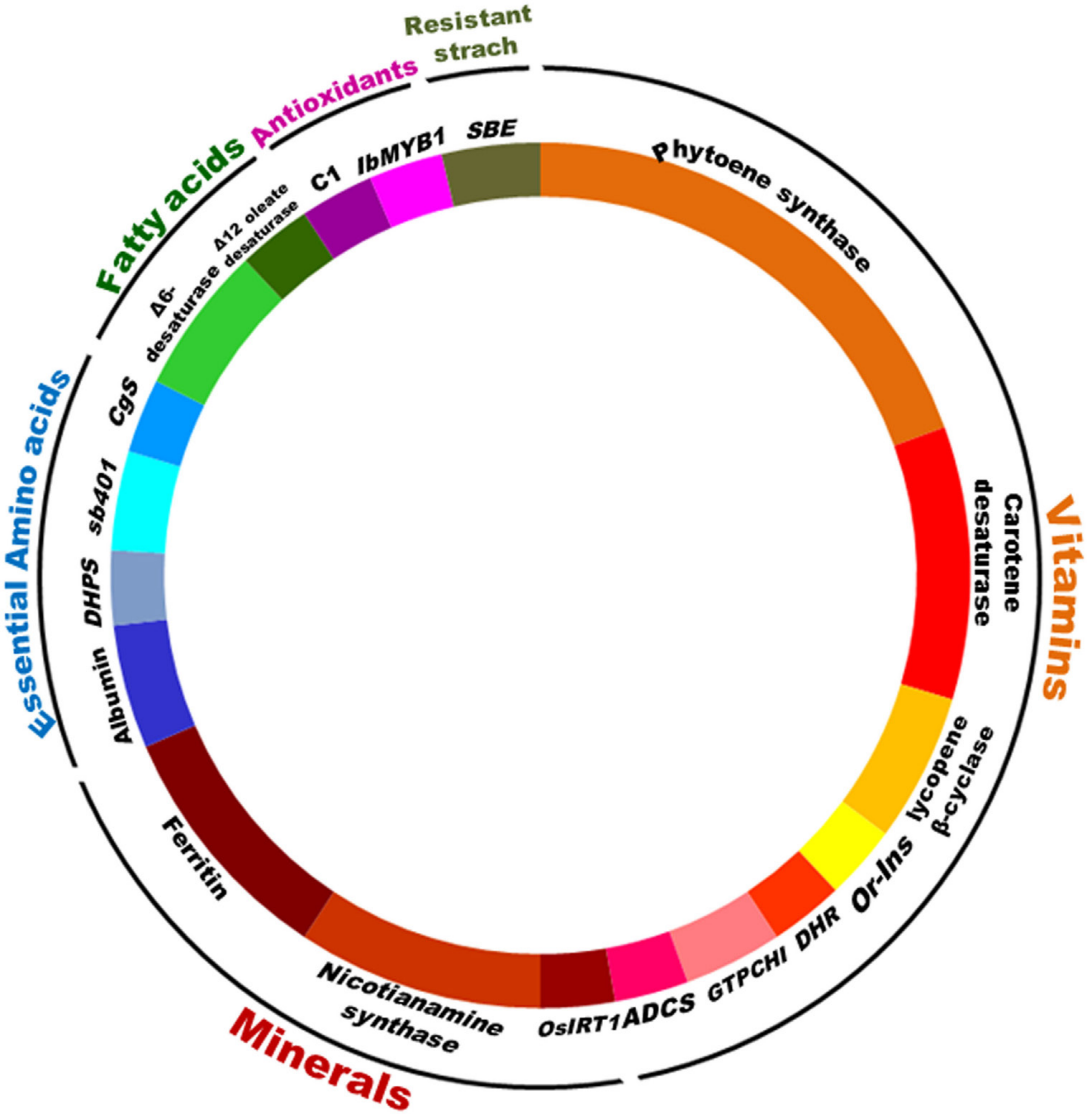
Utilization of different genes for biofortification by transgenic means. Large numbers of genes have been utilized for crop biofortification. Transgenic-based approach has advantages that a useful gene once discovered, can be utilized for targeting multiple crops. Some important genes like phytoene synthase, carotene desaturase, nicotinamide synthase, and ferritin have been utilized in multiple events including multiple crops.

Producing nutritious and safe foods, sufficiently and sustainably, is the ultimate goal of biofortification (34). Biofortification of essential micronutrients into crop plants can be achieved through three main approaches, namely transgenic, conventional, and agronomic, involving the use of biotechnology, crop breeding, and fertilization strategies, respectively. Most of the crops targeted by transgenic, conventional breeding, and agronomical approaches include staple crops like rice, wheat, maize, sorghum, lupine, common bean, potato, sweet potato, and tomato (Figure 2). Cassava, cauliflower, and banana have been biofortified by both transgenic and breeding approaches while barley, soybean, lettuce, carrot, canola, and mustard have been biofortified with transgene and agronomic approaches. Higher numbers of crops have been targeted by transgenic means, while the practical utilization of biofortification is higher by breeding methods (Figures 3A,B). Cereals being staple crop have been targeted by all three approaches. Same is the case of legumes and vegetables. Interestingly, oil seed biofortification has been achieved through transgenic means, because limited availability of genetic diversity for the targeted component, low heritability, and linkage drag in the targeted crop (Figure 3B). Biofortification by breeding has been achieved in crops and specified components when genetic diversity is available in the utilizable form in the primary, secondary, or tertiary gene pool of the targeted crop. When genetic diversity is unavailable, genetic transformation is the better option. Transgenic-based approach has advantages that a useful gene once discovered, can be utilized for targeting multiple crops (Figure 4). Some important genes like phytoene synthase (*PSY*), carotene desaturase, nicotinamide synthase, and ferritin have been utilized in multiple events including multiple crops. In this manuscript, we have compiled the data from research to release on different food crops that are being targeted by the different approaches of biofortification.

## Biofortification through Transgenic Means—Maximum Researched and Minimum Utilized

Transgenic approach can be a valid alternative for the development of biofortified crops when there is a limited or no genetic variation in nutrient content among plant varieties (32, 35). It relies on the access to the unlimited genetic pool for the transfer and expression of desirable genes from one plant species to another which is independent of their evolutionary and taxonomic status. Furthermore, when a particular micronutrient does not naturally exist in crops, transgenic approaches remain the only feasible option to fortify these crop with the particular nutrient (7). The ability to identify and characterize gene function and then utilize these genes to engineer plant metabolism has been a key for the development of transgenic crops (36). Furthermore, pathways from bacteria and other organisms can also be introduced into crops to exploit alternative pathways for metabolic engineering (37).

Transgenic approaches can also be used for the simultaneous incorporation of genes involved in the enhancement of micronutrient concentration, their bioavailability, and reduction in the concentration of antinutrients which limit the bioavailability of nutrients in plants. In addition, genetic modifications can be targeted to redistribute micronutrients between tissues, enhance the micronutrient concentration in the edible portions of commercial crops, increasing the efficiency of biochemical pathways in edible tissues, or even the reconstruction of selected pathways (38–40). Development of transgenically biofortified crops initially involves substantial amount of time, efforts, and investment during research and development stage, but in a long run, it is a cost-effective and sustainable approach, unlike nutrition-based organizational and agronomic biofortification programs (14, 19). Furthermore, genetic engineering has no taxonomic constraints and even synthetic genes can be constructed and used. Transgenic crops with enhanced micronutrient contents hold a potential to reduce micronutrient malnutrition among its consumers, especially poor people in developing countries (12). Numerous crops have been genetically modified to enhance their micronutrient contents. Among micronutrients, vitamins, minerals, essential amino acids, and essential fatty acids have been targeted by the use of various genes from different sources to enhance the food crop nutritional level (Table 2). It has been found that *PSY*, carotene desaturase, and lycopene β-cyclase for vitamins, ferritin and nicotinamine synthase for minerals, albumin for essential amino acids, and Δ^6^ desaturase for essential fatty acids have been widely reported as targets for biofortification (Figure 4). Successful examples of transgenic method are high lysine maize, high unsaturated fatty acid soybean, high provitamin A and iron rich cassava, and high provitamin A Golden rice. Reports are available for biofortified cereals, legumes, vegetables, oilseeds, fruits, and fodder crops.

**Table 2:** Tabulation of crops, nutrients, research status, and concerned publications on biofortification by transgenic means.

Type of cereal	Type of biofortification	Status	Variety/country	Papers
**CEREALS**				
**Rice**				

	Beta-carotenePhytoene (precursor of beta-carotene)	Research		Ye et al. (41); Beyer et al. (42); Datta et al. (43); Paine et al. (44); Burkhardt et al. (45)
	
	Folate (vitamin B9)	Research		Storozhenko et al. (46); Blancquaert et al. (47)
	
	Iron	Research		Takahashi et al. (48); Lee and An (49); Zheng et al. (50); Lee et al. (51); Trijatmiko et al. (52); Goto et al. (53); Vasconcelos et al. (54); Lucca et al. (55); Wirth et al. (56); Masuda et al. (57); Masuda et al. (58)
	
	Phytic acid (iron bioavailability)			Hurrell and Egli (59)
	
	Zinc	Research		Lee and An (49); Masuda et al. (60)
	
	High amino acids and protein content	Research		Zheng et al. (61); Sindhu et al. (62); Lee et al. (63); Katsube et al. (64); Yang et al. (65); Lee et al. (66); Wakasa et al. (67); Zhou et al. (68)
	
	Alpha-linolenic acid	Research		Anai et al. (69)
	
	Flavonoids and antioxidants	Research		Shin et al. (70); Ogo et al. (71)
	
	Resistant starch	Research		Liu et al. (72); Itoh et al. (73); Wei et al. (74)
	
	Human lactoferrin	Research		Nandi et al. (75)

**Wheat**				

	Provitamin ACarotenoids	Research		Wang et al. (76); Cong et al. (77)
	
	Iron	Research		Sui et al. (78); Borg et al. (79)
	
	Phytase or phytic acid	Research		Brinch-Pedersen et al. (80); Bhati et al. (81)
	
	Amino acid composition	Research		Tamás et al. (82)
	
	Anthocyanin	Research		Doshi et al. (83)
	
	Amylose content	Research		Sestili et al. (84)

**Maize**				

	Provitamin ACarotenoids	Research		Aluru et al. (85); Zhu et al. (32); Decourcelle et al. (86)
	
	Vitamin E	Research		Cahoon et al. (87)
	
	Vitamin C	Research		Levine et al. (88); Chen et al. (89)
	
	Multivitamin	Research		Naqvi et al. (90)
	
	Phytase, ferritin (iron bioavailability)	Research		Drakakaki et al. (91); Aluru et al. (92); Chen et al. (93); Shi et al. (94)
	
	Phytate degradation	**Released**	BVLA4 30101 (China)	Origin Agritech (China)
	
	Lysine Lysine and tryptophan Methionine	Research		Yu et al. (95); Tang et al. (96); Frizzi et al. (97); Huang et al. (98); Lai and Messing (99)
	
	Lysine	**Released**	Mavrea™ YieldGard Maize(Japan, Mexico) Mavera™ Maize (LY038) (Australia, Columbia, Canada, Japan, Mexico, New Zealand, Taiwan, USA)	Monsanto Renessen LLC (Netherland)
	
	Human lactoferrin	Research		Yang et al. (40)

**Barley**				

	Zinc	Research		Ramesh et al. (100)
	
	Phytase	Research		Holme et al. (101)
	
	Lysine			Ohnoutkova et al. (102)
	
	Beta-glucan	Research		Dikeman and Fahey (103); Burton et al. (104)
	
	Resistant starch	Research		Carciofi et al. (105)
	
	Polyunsaturated fatty acids	Research		Mihalik et al. (106)
	
	Human lactoferrin	Research		Kamenarova et al. (107)

**Sorghum**				

	Provitamin A	Research		Lipkie et al. (108)
	
	Lysine	Research		Zhao et al. (109)
	
	Improved protein digestibility	Research		Elkonin et al. (110); Grootboom et al. (111)
**LEGUMES/PULSES**				
**Soybean**				

	Beta-carotene	Research		Schmidt et al. (112); Pierce et al. (113); Kim et al. (114)
	
	Vitamin E	Research		Van Eenennaam et al. (115)
	
	Cysteine	Research		Kim et al. (116)
	
	Methionine and cysteineMethionine			Dinkins et al. (117); Song et al. (118); Hanafy et al. (119)
	
	Linoleic acid γ-Linolenic Acid + stearidonic acid (STA)	Research		Flores et al. (120); Sato et al. (121)
	
	STA	Research		Eckert et al. (122)
	
	Oleic acid	Research		Zhang et al. (123)
	
	Arachidonic acid	Patent		Patent-US 7943816 B2
	
	Flavonoids	Research		Yu et al. (124)
	
	Oleic acid	**Released**	G94-1, G94-19, G16 (Australia, Canada, Japan, New Zealand, USA) Treus™, Plenish™ (DP305423; Australia, Canada, China, Japan, Mexico, Philippines, Singapore, South Africa, South Korea, Taiwan and USA) Treus™ (DP 305423 × GTS 40-3-2; Argentina, Canada, China, Japan, Mexico, Philippines, South Africa, South Korea, Taiwan) MON 87705 × MON 89788(European Union, Mexico, South Korea, Taiwan)	Dupont
			Vistuve Gold™ (MON87708; Australia, Columbia, Canada, European Union, Indonesia, Japan, Mexico, New Zealand, Philippines, Singapore, South Korea, Taiwan, USA, Vietnam) MON87705 × MON87708 × MON89788 andMon87705 × MON87708 × MON89788 (Canada)	Monsanto
	
	STA	**Released**	MON 87769 × MON 89788(Mexico, South Korea, Taiwan) MON87769(Australia, Columbia, Canada, European Union, Indonesia, Japan, Mexico, New Zealand, Philippines, South Korea, Taiwan, USA, Vietnam)	Monsanto

**Common bean**				

	Methionine	Research		Aragao et al. (125)

**Lupines**				

	Methionine	Research		Molvig et al. (126)
**VEGETABLES**				
**Potato**				

	Beta-caroteneZeaxanthin	Research		Ducreux et al. (127); Diretto et al. (128); Van Eck et al. (129); Song et al. (130); Lopez et al. (131); Romer et al. (132)
	
	Ascorbate	Research		Hemavathi et al. (133)
	
	Methionine	Research		Dancs et al. (134); Huang et al. (135); Zeh et al. (136); Goo et al. (137); Di et al. (138)
	
	Amino acid composition	Research		Chakraborty et al. (139)
	
	Cyclodextrins (carbohydrate)	Research		Oakes et al. (140)
	
	Anthocyanins + phenolic acids	Research		Lukaszewicz et al. (141)
	
	Fructan and inulin	Research		Hellwege et al. (142); Hellwege et al. (143)
	
	Reduced amylose and increased amylopectin in starch granules	**Released**	Starch Potato (AM 04—1020)(USA)	BASFBASF
			Amflora™ (EH 92-527-1)(European Union)	BASF

**Sweet potato**				

	Beta-carotene	Research		Kim et al. (144)
	
	Antioxidants	Research		Park et al. (145)

**Cassava**				

	Beta-caroteneProvitamin A	Research		Telengech et al. (146); Welsch et al. (147)
	
	Iron	**Released**		Biocassava Plus
	
	Beta-carotene	**Released**		Biocassava Plus
	
	Protein	**Released**		Biocassava Plus

Carrot	Ca	Research		Park et al. (148); Morris et al. (149)

Lettuce	Iron	Research		Goto et al. (150)

Cauliflower	Beta-carotene	Research		Lu et al. (151)
**OILSEED**				
**Linseed/flax**				

	Increased flavonoid content	Research		Lorenc-Kukula et al. (152)
	
	Very long-chain polyunsaturated fatty acids accumulation	Research		Galili et al. (153); Abbadi et al. (154)
	
	Carotenoids in Flaxseed (*Linum usitatissimum*)	Research		Fujisawa et al. (155)
	
	Essential amino acids	**Released**	CDC Triffid Flax (FP967) (Canada, Colombia, USA)	University of Saskatchewan, Canada

**Canola**				

	Beta-carotenes and its precursors	Research		Shewmaker et al. (38); Ravanello et al. (156); Fujisawa et al. (157); Yu et al. (158); Wei et al. (159)
	
	Lysine	Research		Falco et al. (160)
	
	Fatty acid composition	Research		Dehesh et al. (161)
	
	γ-Linolenic acid	Research		Liu et al. (162); Flider (163)
	
	Phytate degradation (increase in available P)	**Released**	Phytaseed™ Canola (MPS 961) (USA)	BASF
	
	Phytate degradation (increase in available P)	**Released**	Phytaseed™ Canola (MPS 962) (USA)	BASF
	
	Phytate degradation (increase in available P)	**Released**	Phytaseed™ Canola (MPS 963) (USA)	BASF
	
	Phytate degradation (increase in available P)	**Released**	Phytaseed™ Canola (MPS 964) (USA)	BASF
	
	Phytate degradation (increase in available P)	**Released**	Phytaseed™ Canola (MPS 965) (USA)	BASF

**Mustard**				

	γ-linolenic acid	Research		Hong et al. (164)
**FRUITS**				
**Tomato**				

	Folate, phytoene, Beta-carotene, lycopene, provitamin A, IsoprenoidsCarotenoid + flavonoid	Research		Enfissi et al. (165); Fraser et al. (166); Rosati et al. (167); Apel and Bock (168); Wurbs et al. (169); Huang et al. (170); Dharmapuri et al. (171); Davuluri et al. (172)
	
	Ascorbate	Research		Zhang et al. (173); Haroldsen et al. (174); Cronje et al. (175); Chen et al. (89)
	
	Folate	Research		De la Graza et al. (176); De la Graza et al. (177)
	
	Antioxidant anthocyanins and its precursors	Research		Muir et al. (178); Zuluaga et al. (179); Niggeweg et al. (180); Giovinazzo et al. (181); Luo et al. (182); Shih et al. (183)

**Apple**				

	Stilbenes	Research		Szankowski et al. (184)

**Banana**				

	Beta-carotene	Research		Waltz (185)
**FODDER**				
**Alfalfa**				

	Isoflavonoids	Research		Deavours et al. (186)
	
	Methionine	Research		Avaram et al. (187)
	
	Low lignin	Research		Reddy et al. (188)
	
	Phytase	Patent		Austin-Phillips et al. (189) (US 6248938 B1)

*Significant amount of information have been generated that hold a bright future to address the malnutrition challenge*.

## Transgenic Cereals

### Transgenic Rice (*Oryza sativa*)

Rice has been targeted to address the global challenge of undernutrition. Vitamin deficiency is one of the major challenges that affect underprivileged population due to poor affordability. Golden Rice was an important breakthrough in this direction as an effective source of provitamin A (beta-carotene) with a significant potential to reduce disease burden by expressing genes encoding *PSY* and carotene desaturase (41–45). The level of beta-carotene precursor, i.e., phytoene, has been enhanced up to 23-fold by targeting gene encoding carotene desaturase (45). Folic acid (vitamin B9) is important for normal pregnancy and anemia (190). Rice has been genetically modified to increase folate content (up to 150-fold) by overexpressing genes encoding *Arabidopsis* GTP-cyclohydrolase I (GTPCHI) and aminodeoxychorismate synthase [ADCS (46, 47)]. The 100 g of modified rice was found to be sufficient to meet daily folate requirements of an adult individual.

Rice has also been targeted to address the global challenge of iron deficiency anemia. Multiple reports have indicated an increase in iron content in rice by expressing genes encoding, nicotianamine aminotransferase (48), iron transporter *OsIRT1* (49), nicotianamine synthase 1 (*OsNAS1*) and 2 (*OsNAS2*) (50–52, 191), soybean ferritin (52–54), and common bean ferritin (55). Iron biofortified rice was also synthesized by introducing multiple genes involved in iron nutrition (56–58). In addition to enhanced iron content, improvement in iron bioavailability was also achieved by reducing antinutrient compounds in rice such as phytic acid (59). Similarly, zinc content was also elevated in GM rice by overexpressing *OsIRT1* (49) and mugineic acid synthesis genes from barley [*HvNAS1, HvNAS1, HvNAAT-A, HvNAAT-B, IDS3* (60)].

Improvement in quality protein has been addressed by targeting essential amino acid content in rice by expressing seed-specific genes of bean β-phaseolin (61), pea legumin (62); Sesame 2S Albumin (63); soybean glycinin (64); bacterial aspartate kinase, dihydrodipicolinate synthase (DHPS) (65); maize DHPS (66); rice anthranilate synthase α-subunit (67); and *E. coli* aspartate aminotransferase (68). Rice has also been targeted for seed oil quality by increasing amount of polyunsaturated fatty acid that can help in the reduction of bad cholesterol levels in the body and improve human nutrition (192). An essential fatty acid α-linolenic acid has been enhanced in rice by expressing soybean omega-3 fatty acid desaturase (FAD3) gene [*GmFAD3* (69)]. Flavonoids are associated with antioxidant activity and its content in rice has been enhanced by expressing maize C1 and R-S regulatory genes [Myb-type and basic helix-loop-helix-type transcription factors (70)]; and phenylalanine ammonia lyase and chalcone synthase (*CHS*) genes (71). To address the challenge of overnutrition and obesity, the content of less digestible and resistant amylose starch has been enhanced by expression of antisense waxy genes (72, 73) and antisense RNA inhibition of starch-branching enzymes (SBE) (74). Besides introducing micronutrients, expression of functional human milk protein (lactoferrin) in rice grains has opened the possibility for creating a value-added cereal-based ingredients that can be introduced into infant formula and baby food (75, 193).

### Transgenic Wheat (*Triticum aestivum*)

Wheat is one of the most widely grown staple food crops in the world. Researchers have tried to address the challenges of most deficient nutrients like vitamin A, iron, and quality proteins through wheat. The provitamin A content of wheat has been enhanced by expressing bacterial *PSY* and carotene desaturase genes [*CrtB, CrtI* (76, 77)]. The iron content in wheat has been enhanced by expression of ferritin gene from soybean (78) and wheat [*TaFer1-A* (79)]. To increase iron bioavailability phytase activity was increased by the expression of the phytochrome gene [*phyA* (80)] and phytic acid content has been decreased by silencing of wheat ABCC13 transporter (81). Protein content, especially essential amino acids lysine, methionine, cysteine, and tyrosine contents of wheat grains were enhanced using Amaranthus albumin gene [*ama1* (82)]. Wheat has also been targeted to improve the antioxidant activity by expressing maize regulatory genes (*C1, B-peru*) involved in anthocyanin production (83). To address the challenge of overnutrition and obesity, the content of less digestible and resistant amylose starch has been enhanced by silencing gene encoding SBE [*SBEIIa* (84)].

### Transgenic Maize (*Zea mays*)

Maize is one of the important staple crops in developing countries, and it has been addressed for vitamins, minerals, quality protein, and antinutrient components by means of genetic engineering. Maize endosperm has been enriched with provitamin A (carotenoids) by expressing bacterial *crtB* (85) and multiple (5) carotenogenic genes (86, 194). Vitamin E and its analog are potent antioxidants with implications over human health and many research groups are emphasizing on biofortification of these components in maize crop. Tocotrienol and tocopherol content in maize has been increased by overexpression of homogentisic acid geranylgeranyl transferase [HGGT (87)]. Vitamin C (l-ascorbic acid) a water-soluble antioxidant play roles in cardiovascular function, immune cell development, and iron utilization (88). Its level in corn has been enhanced nearly 100-fold times by recycling oxidized ascorbic acid to reduced form by the expression of dehydroascorbate reductase [DHAR (89)]. On the other hand, Naqvi et al. (90) developed multivitamin corn containing 169-fold the normal amount of beta-carotene, double the normal amount of folate and 6-fold the normal amount of ascorbate by engineering three distinct metabolic pathways.

Bioavailability of micronutrients is hindered by antinutrient components. Bioavailability of iron has been increased by expressing soybean ferritin and *Aspergillus* phytase (91), soybean ferritin (92), *Aspergillus niger phyA2* (93), and silencing the expression of ATP-binding cassette transporter and multidrug resistance-associated protein (94). As a practical example, BVLA4 30101 variety released by Origin Agritech in China has been biofortified for phytate degradation.

The major maize seed storage proteins, zeins have poor nutritional quality due to lower content of essential amino acids lysine and tryptophan. In maize essential amino acid content has been targeted with significant achievement. Lysine content in maize has been increased by expression of *sb401* from potato (95, 96), single bifunctional expression/silencing transgene cassette (97). Both lysine and tryptophan content have been increased in maize by antisense dsRNA targeting alpha-zeins [both 19- and 22-kDa (98)]. Importance of lysine content in maize is evident from maize varieties rich in lysine *viz*., Mavrea™YieldGard Maize that has been released by Monsanto in Japan and Mexico; Mavera™ Maize (LY038) by Renessen LLC (Netherland) in Australia, Columbia, Canada, Japan, Mexico, New Zealand, Taiwan, USA The amino acid methionine is a common protein building block that is also important in other cellular processes. Its content has been increased in maize by modifying *cis*-acting site for *Dzs10* (99). Amino acid balance of maize has also been improved by expressing milk protein α-lactalbumin (40).

### Transgenic Barley (*Hordeum vulgare*)

Barley being a model cereal crop has been targeted to improve its micronutrient content. Its zinc content has been improved by overexpression of zinc transporters (100). To increase the bioavailability of iron and zinc, phytase activity has been increased in barely seeds by expression of phytase gene [*HvPAPhy*_a (101)]. Essential amino acid lysine has been enhanced in barley by expressing DHPS gene [*dapA* (102)]. β glucans are dietary fibers and are believed to dramatically reduce the risk of contracting serious human diseases such as cardiovascular disease and type II diabetes (103). Its content has been increased in barley by overexpression of cellulose synthase-like gene [*HvCslF* (104)]. Resistant starch (amylose only) barley has been produced by the RNAi approach by suppressing all genes coding for SBE [*SBE I, SBE IIa, SBE IIb* (105)]. Content of health promoting polyunsaturated fatty acids, γ-linolenic acid, and stearidonic acid (STA) has been improved in barley by expressing Δ^6^-desaturase [*D6D* (106)]. Barley has been targeted to express human lactoferrin gene [*HLF* (107)]. Apart from this several medicinally and industrially important bioactives including enzymes and antibiotics have been expressed in barley.

### Transgenic Sorghum (*Sorghum bicolor*)

Sorghum is one of the most important staple foods for millions of poor rural people. It has an ability to grow well in harsh environments. It has been targeted to improve provitamin A (beta-carotene) by expressing *Homo188-A* (108). Content of essential amino acid lysine has been improved in sorghum by the introduction of a high lysine protein [HT12 (109)]. One of the issues with sorghum consumption is that its grains are less digestible than the other major staple crops. Its seed storage proteins, γ-kafirin, is resistant to protease digestion. Digestibility index of transgenic sorghum has been increased by RNAi silencing of the *γ-kafirin* (110) and combined suppression involving three genes [*γ-kafirin-1, γ-kafirin-2*, and α*-kafirin A1* (111)].

## Transgenic Legumes and Pulses

### Transgenic Soybean (*Glycine max*)

Soybean is a global source of vegetable oil and high-quality protein. The soybean has been targeted to increase provitamin A (beta-carotene), a monounsaturated ω-9 fatty acid (oleic acid) and seed protein contents by expressing bacterial *PSY* gene (112). In a different approach provitamin A (Canthaxanthin) was enhanced by expressing bacterial *PSY* [*crtB, crtW, bkt1* (113)]. Kim et al. (114) has demonstrated the production of a high provitamin A (beta-carotene) soybean through overexpression of *PSY* and carotene desaturase. Another important nutrient vitamin E activity in barley has been enhanced with increased content of δ-tocopherol and decreased γ-tocopherol by coexpressing 2-methyl-6-phytyl benzoquinol methyltransferase genes [*At-VTE3*; *At-VTE4* (115)]. Soybeans contain approximately 40% protein, but they are deficient in one or more of the essential amino acids, especially the sulfur-containing amino acids, cysteine and methionine. The cysteine content of soybean seeds has been increased through overexpression of the sulfur assimilatory enzyme, O-acetylserine sulfhydrylase (116). Similarly, Dinkins et al. (117) increased methionine and cysteine content in soybean by overexpressing the maize zein protein. The methionine content of soybean has been increased by expressing cystathionine γ-synthase (118, 119). Soybean is rich in healthy oil and has approximately 20% oil content. But 7–10% of the oil contains unstable fatty acid α-linolenic acids that contribute to reduced soybean seed oil quality. It results in the formation of undesirable *trans*-fatty acid as a result of hydrogenation (195). To enhance the agronomic value of soybean seed oil by reducing the levels of α-linolenic acids (18:3), siRNA-mediated gene silencing-based approach has been utilized for silencing of ω-3 FAD3 (120). In another experiment γ-linolenic acid (GLA) and STA (ω-3 fatty acids) content in soybean oil has been increased by expression of Δ^6^-desaturase gene that is responsible for the conversion of linoleic acid and α-linolenic acid to GLA and STA (121). Similarly, STA content has been increased by simultaneous expression of Δ^6^ desaturase and Δ^15^ desaturase (122). Antisense RNA technology has been used to reduce the amount of linoleic acid and palmitic acid and increase the amount of oleic acid by inhibition of expression of Δ^12^ oleate desaturase [*GmFAD2-1b* (123)] that converts oleic acid into linoleic acid. Soybean seeds are low in isoflavone content. Consumption of isoflavone is associated with human health benefits such as decreased risk of heart disease, reduced menopausal symptoms, and reduced risk of some hormone-related cancers (196). Isoflavone content has been enhanced in soybean seeds by the combination of maize C1 and R transcription factor-driven gene activation and suppression of a competing pathway (124).

Importance of improvement in ω-3 fatty acid content in soybean is evident from the fact that a large number of cultivars with improved oleic, linoleic, and STA have been released by private companies. Transgenic soybean varieties rich in oleic acid *viz*., G94-1, G94-19, G168 have been released in Australia, Canada, Japan, New Zealand, USA; and Treus™, Plenish™ (DP305423) in Australia, Canada, China, European Union, Japan, Mexico, New Zealand, Philippines, Singapore, South Africa, South Korea, Taiwan, USA; and Treus™ (DP 305423 × GTS 40-3-2) in Argentina, Canada, China, Japan, Mexico, Philippines, South Africa, South Korea, Taiwan by Dupont. The transgenic varieties of soybean rich in oleic acid were released by Monsanto, *viz*., Vistive Gold™ (MON87705) in Australia, Columbia, Canada, European Union, Indonesia, Japan, Mexico, New Zealand, Philippines, Singapore, South Korea, Taiwan, USA, Vietnam; MON87705 × MON87708 × MON89788 and MON 87705 × MON 87708 × MON 89788 in Canada. The soybean variety rich in oleic acid and linoleic acid was released in the European Union, Mexico, South Korea, and Taiwan. The other varieties rich in STA *viz*., MON 87769 × MON 89788 were released in Mexico, South Korea, Taiwan and MON87769 released in Australia, Columbia, Canada, European Union, Indonesia, Japan, Mexico, New Zealand, Philippines, South Korea, Taiwan, USA, Vietnam by Monsanto company.

### Transgenic Common Beans (*Phaseolus vulgaris*)

The common bean is among the most important grain legumes used for human consumption. However, although beans are rich in some essential amino acids, e.g., lysine, threonine, valine, isoleucine, and leucine, their nutritional value is limited because of the small amounts of the essential amino acid methionine and cysteine. Common bean methionine content has been increased by the expression of methionine-rich storage albumin from Brazil nut (125).

### Transgenic Lupines (*Lupinus angustifolius*)

Lupine is the major grain legume. The lupine seed protein, in common with the protein of most other grain legumes, is deficient in the sulfur-containing amino acids methionine and cysteine. Its methionine content has been increased by the expression of sunflower seed albumin gene (126).

## Transgenic Vegetables

### Transgenic Potato (*Solanum tuberosum*)

Potato is the world’s fourth most important source of calories, and it’s any nutritional enhancement is of great significance. In potato tuber, provitamin A (carotenoid forms) have been increased by incorporating *PSY* gene (127) and by simultaneous incorporation of three genes: *PSY*, phytoene desaturase, and lycopene β-cyclase (128). Beta-carotene content in tubers has been also enhanced by using RNAi to silence the beta-carotene hydroxylase gene (bch), which converts beta-carotene to zeaxanthin (129) and by regulation of beta-carotene synthesis through expression of lycopene β-cyclase [*StLCYb* (130)]. In another experiment, it has been observed that incorporation of *Or* gene from orange cauliflower mutant leads to increase in carotenoids along with three additional metabolite intermediates phytoene, phytofluene, and z-carotene (131). Zeaxanthin which is another form of carotenoid has been also increased by expressing zeaxanthin epoxidase genes in transgenic potato tuber (132).

The potato has been also targeted for enhancement of vitamin C (ascorbic acid) by overexpressing strawberry *GalUR* (133). Potato tubers are very poor in essential amino acid, methionine, which has been targeted for its enhancement by coexpressing cystathionine γ-synthase (*CgSΔ*_90_) and methionine-rich storage protein (134). Similarly, silencing of *StMGL1* (135) and antisense inhibition of threonine synthase (136) led to increase in methionine to isoleucine ratio and methionine content (up to 239-folds) in potato tubers. Methionine content has been also enhanced by overexpressing the gene encoding the seed storage protein from *Perilla* [PrLeg polypeptide (137)] and cystathionine γ-synthase (*CgS*) genes (138). Transgenic potatoes expressing Amaranth albumin (*ama1*) result in an increase in total protein content in tubers along with the significant increase in the concentration of several essential amino acids including methionine (139).

High value carbohydrate rich potato tubers has been synthesized by expressing cyclodextrin glycosyltransferases (*CGT*) gene, which results in the production of multipurpose dietary fiber cyclodextrins from starch (140). Potato tubers have been also focused upon to increase the phenolic acid, and anthocyanins contents by the single-gene overexpression or by simultaneous expression of *CHS*, chalcone isomerase (*CHI*), and dihydroflavonol reductase (141). It has been also targeted to improve the content of dietary fiber fructan and inulin (142, 143). Transgenic potato varieties engineered for starch quality, which has reduced amylose and increased amylopectin in starch granules were released by BASF *viz*., Starch Potato (AM 04—1020) in the USA and Amflora™ (EH 92-527-1) in the European Union. Transgenic potato varieties that limit formation of the reducing sugars through starch degradation have been released in Canada and USA by J. R. Simplot Co.

### Transgenic Sweet Potato (*Ipomea batatas*)

Sweet potato is an alternative source of bioenergy and natural antioxidants. It is rich in various phytochemicals, anthocyanins, vitamin C, carbohydrates, potassium, and dietary fiber (197). Its nutrition properties have been further enhanced by increasing the contents of carotene, lutein, and total carotenoids by overexpressing orange *IbOr-Ins* gene in white fleshed sweet potato (144). The antioxidant capacity of orange-fleshed sweet potato cultivar has been increased by overexpression of *IbMYB1* a key regulator of anthocyanin biosynthesis in the storage roots (145).

### Transgenic Cassava (*Manihot esculenta*)

Cassava is an important staple food crop for millions of poor people worldwide as it is tolerant to different stresses. However, cassava is deficient in several important nutrients like provitamin A, vitamin E, iron, and zinc. Cassava biofortification of provitamin A, iron, and zinc has been carried out to reduce their deficiency among the undernourished communities. Telengech et al. (146) as a part of the BioCassava Plus project developed transgenic cassava that expresses beta-carotene in roots using *npt*II, *crtB*, and *DXS*. Similarly, Welsch et al. (147) showed that the cassava plants overexpressing a *PSY* transgene produced yellow-fleshed, high-carotenoid roots. Different transgenic cassava varieties biofortified for enhanced levels of iron, beta-carotene, and zinc are under development and field trials in the Biocassava Plus Program targeted at African countries.

### Transgenic Carrot (*Daucus carota* subsp. *sativus*)

Carrots are one of the most popular vegetables and contain high levels of beta-carotene and vitamins and minerals; however, like many vegetables, these are poor in calcium content (198). Bioavailable calcium content in transgenic carrot has been increased by expressing the *Arabidopsis* H^+^/Ca^2+^ transporter [CAX1 (148, 149)].

### Transgenic Lettuce (*Lactuca sativa*)

Lettuce is one of the most popular leafy vegetables all around the world. Compared to spinach, the iron content of lettuce is low. The lettuce has been improved for iron content, yield, and growth rate by expressing a soybean ferritin gene (150).

### Transgenic Cauliflower (*Brassica oleracea*)

Cauliflower is a popular vegetable in several parts of the world. It is rich in antioxidant phytonutrients. Its nutritional value has been further enhanced by increasing beta-carotene content in mutant orange cauliflower by the insertion of a copia-like LTR retrotransponson in the *Or* (151).

## Transgenic Oilseeds

### Transgenic Linseed (*Linum usitatissimum*)

Linseed edible oil is in demand as a nutritional supplement. Linseed or flax seeds are the richest source of polyunsaturated fatty acids, but linseed oil is highly susceptible to auto-oxidation, which generates toxic derivatives. Genetically modified flax plants with increased antioxidant potential, stable, and healthy oil production has been generated by suppressing *CHS* gene that resulted in hydrolyzable tannin accumulation (152). Very long-chain unsaturated fatty acids (VLCPUFA) are important fatty acids with limited supply due to decrease in marine resources such as fish oils. It can be compensated by implementation of VLCPUFA biosynthesis into oilseed crops (153). VLCPUFA such as arachidonic acid (C20:4 n-6), eicosapentenoic acid (EPA C20:5 n-3), and docosahexenoic acid (DHA C22:5 n-3) are considered to be nutritionally beneficial because of their function as cholesterol-lowering agents (199). Researchers have intended to enhance the accumulation of Δ^6^ desaturated C18 fatty acids and C20 polyunsaturated fatty acids, including arachidonic and eicosapentaenoic acid by seed-specific expression of cDNAs encoding fatty acyl-desaturases and elongases in linseed (154). Enrichment of carotenoids in flaxseed has been done by the introduction of *PSY* gene [*crtB* (155)]. Transgenic linseed rich in essential amino acids *viz*., CDC Triffid Flax (FP967) has been released by University of Saskatchewan, in Colombia, USA, and Canada.

### Transgenic Canola (*Brassica napus*)

Canola is an important oilseed crop for millions of people around the world. Canola produces edible oil lower in saturated fat and higher in omega-3 fatty acids. To further enhance its health benefits its carotenoid content (mainly alpha and beta-carotenes) has been increased by overexpressing bacterial *PSY* [crtB (37)]. Higher β-carotenoid content has been achieved by simultaneous expression of *PSY*, phytoene desaturase, and lycopene cyclase genes (155) and simultaneous expression of seven bacterial genes; *idi, crtE, crtB, crtI, crtY, crtW*, and *crtZ* (157). Higher beta-carotene content along with high xanthophylls and lutein contents have been achieved by RNAi silencing of lycopene ε-cyclase [ε-*CYC* (158)] and DET1 (159). Essential amino acid lysine has been increased in canola by expression of aspartokinase (AK) and dihydrodipicolinic acid synthase (DHDPS) genes (160). Increase in level of two fatty acids *viz*., caprylate (8:0) and caprate (10:0) in canola seed oil accompanied by a preferential decrease in the levels of linoleate (18:2) and linolenate (18:3) has been achieved by overexpression of thioesterase gene [Ch FatB2 (161)]. Canola normally does not have any Δ^6^ desaturase activity and thus lack GLA. In order to produce GLA more economically and to make it more readily available transgenic lines rich in GLA has been developed by expression of Δ^12^ or Δ^6^ desaturases genes (162, 163). Phytic acid is known as a food inhibitor, which chelates micronutrient and prevents its bioavailability, as human and other monogastic animals lack the phytase enzyme in their digestive track. Transgenic canola varieties *viz*., Phytaseed™ Canola (MPS 961-965) engineered for phytase degradation to enhance the availability of phosphorus in canola has been produced and released by BASF in USA.

### Transgenic Mustard (*Brassica juncea*)

Mustard is an economically significant crop and extensively cultivated for oil throughout the world. It has been targeted for improving the nutritionally important unsaturated fatty acids. This has been achieved by the expression of the enzyme Δ^6^ FAD3 that led to the production of gamma linoleic acid in the transgenic mustard (164).

## Transgenic Fruits

### Transgenic Tomato (*Solanum lycopersicum*)

Tomato is one of the most popular fruits, consumed by billions around the world and is an important source of vitamin C, micronutrients, and other phytonutrients. It derives its color from isopernoid lycopene. Isoprenoids are one of the largest classes of natural products with several thousand compounds. In higher plants, isoprenoids have essential roles in membrane structure (sterols), free radical scavenging (carotenoids and tocopherols), redox chemistry (plastoquinone, ubiquinone), defense mechanisms (phytoalexins), and growth regulation (gibberellins, cytokinins, brassinosteroids, and abscisic acid) (200). Several attempts have been made to increase the isoprenoid content in tomato. The sterol content was elevated in tomato by expression of 3-hydroxymethylglutaryl CoA [*hmgr-1* (165)]. Tomato phytoene and beta-carotene content has been enhanced by expression of 1-deoxy-d-xylulose-5-phosphate synthase [*dxs* (165)]. Higher contents of lycopene, beta-carotene, and lutein have also been achieved in tomato by the expression of *PSY* gene [*crtB* (166)]. Double biofortification of carotenoid and flavonoid contents have also been achieved by RNAi technology by suppressing photomorphogenesis regulatory gene [*DET1* (172)]. The beta-carotene content has also been increased by overexpression of lycopene beta-cyclase gene [*beta-Lcy* (167–169)]. Higher contents beta-carotene as well as its hydroxylation product xanthophylls (beta-cryptoxanthin and zeaxanthin) has been obtained by simultaneous expression of *beta-Lcy* and beta-carotene hydroxylase [b-Chy (171)]. Total carotenoid and high value astaxanthin content (hydroxylation product of a beta-carotene) have been enhanced in tomato by expression of beta-carotene ketolase and hydroxylase (170). The tomato has been targeted to improve its vitamin C (ascorbic acid) content by overexpressing GDP-mannose 3′,5′-epimerase [*SlGME1, SlGME2* (173)], DHAR (174), and coexpression of three genes GDP-mannose pyrophosphorylase, arabinono-1,4-lactone oxidase, and myo-inositol oxygenase 2 (88, 175). Another important nutrient folic acid has been targeted by overexpression of GTPCHI (176) and aminodeoxychorismate synthase (177).

Tomato has also been selected to increase antioxidant anthocyanins by expression of *CHI* (178), transcriptional activators *AtMYB75* (179), and expression of two transcription factors, *Delila* and *Rosea1* (201). Other antioxidants like chlorogenic acid have been targeted by gene silencing of HQT (180), trans-resveratrol by expression of stilbene synthase (181), polyphenolic antioxidants by expression of AtMYB12 (182), and genistin by overexpression of isoflavone synthase (IFS) gene (183). Anthocynin rich blue transgenic tomato has been developed by Norfolk plant sciences.

### Transgenic Apple (*Malus domestica*)

Apple has long been recognized as a great source of antioxidants. Apple has been bioengineered with a stilbene synthase gene from the grapevine (*Vitis vinifera* L.) thereby leading to synthesis of resveratrol in transgenic apple, thereby, expanding the antioxidant capacity (184).

### Transgenic Banana (*Musa acuminata*)

The banana, a fourth most important food crop of the developing countries, has been predominantly targeted for beta-carotene. This has been achieved by developing transgenic banana (Super Banana) by expressing *PSY* gene (*PSY2a*) of Asupina banana, which is naturally high in beta-carotene (185).

## Transgenic Fodder

### Transgenic alfalfa (*Medicago sativa*)

Alfalfa is as an important feed legume crop in many countries. Attempts have been made to improve its nutritional status through enhancement of isoflavonoids, essential amino acids, and improve its digestibility. Isoflavonoids are a predominantly legume-specific subclass of flavonoid secondary metabolites. Transgenic alfalfa has been generated by constitutively expressing IFS that is correlated with its increased isoflavonid composition (186). Alfalfa suffers from a limited level of the sulfur-containing amino acids, methionine, and cysteine. Its methionine content has been increased by the expression of cystathionine γ-synthase [*AtCgS* (187)]. Improvement in the digestibility of forages has also been an area of interest as it correlates with animal performance. By targeting three specific cytochrome P450 enzymes for antisense downregulation, transgenic alfalfa lines have been generated with low lignin content (188). Alfalfa has also been engineered to increase phytase activity, and thereby enabling its use in animal feeds, including livestock, poultry, and fish feed (189).

## Biofortification through Agronomic Approaches

Biofortification through agronomic methods requires physical application of nutrients to temporarily improve the nutritional and health status of crops and consumption of such crops improves the human nutritional status (202). In comparison with inorganic forms of minerals, the organic ones are more available for a man, as they can be absorbed more easily; and are less excreted (203) and their toxicity symptoms are less intensive (DRI 2000). It generally relies on the application of mineral fertilizers and/or increase in their solubilization and/or mobilization from the soil in the edible parts of plants. Macrominerals like nitrogen, phosphorus, and potassium (NPK) make an important contribution to the attainment of higher crop yields (204). Through the application of NPK-containing fertilizers, agricultural productivity increased in many countries of the world in the late 1960s and resulted in Green Revolution and saved them from starvation. In the current scenario, these fertilizers are important and necessary to improve crop yield and save the human population from starvation as low-input agriculture cannot feed the current seven billion world population (205). Microminerals iron, zinc, copper, manganese, I, Se, Mo, Co, and Ni are found in varying degrees in the edible portion of certain plants and are usually absorbed from the soil. Improvement of the soil micronutrient status by their application as fertilizers can contribute to decrease in micronutrient deficiency in humans (206). When crops are grown in soils, where mineral elements become immediately unavailable in the soil and/or not readily translocated to edible tissues targeted application of soluble inorganic fertilizers to the roots or to the leaves are practiced. Agronomic biofortification is simple and inexpensive, but needs special attention in terms of source of nutrient, application method and effects on the environment. These should be applied regularly in every crop season and thus are less cost-effective in some cases. Use of mineral fertilizers is evidently feasible in the developed world, as exemplified by the success of Se fertilization of crops in Finland (207), zinc fertilization in Turkey (208), and I fertilization in irrigation water in China (209).

In addition to fertilizers, plant growth-promoting soil microorganisms can be used to enhance the nutrient mobility from soil to edible parts of plants and improve their nutritional status. Soil microorganisms like different species of genera *Bacillus, Pseudomonas, Rhizobium, Azotobacter*, etc. can also be utilized to increase the phytoavailability of mineral elements (210, 211). The N_2_-fixing bacteria play important role in increasing crop productivity in nitrogen limited conditions (212). Many crops are associated with mycorrhizal fungi that can release organic acids, siderophores, and enzymes capable of degrading organic compounds and increasing mineral concentrations in edible produce (210, 213). Different crops have been targeted through agronomical biofortification to improve the human nutritional status (Table 3).

**Table 3:** Tabulation of crops, nutrients, research status, and concerned publications on biofortification through agronomic approaches.

Type of cereal	Type of biofortification	Status	Papers
**CEREALS**			
**Rice**			

	Iron	Research	He et al. (214); Yuan et al. (215); Fang et al. (216); Wei et al. (217); Yuan et al. (215)
	
	Zinc	Research	Wei et al. (218); Boonchuay et al. (219); Jiang et al. (220); Mabesa et al. (221); Shivay et al. (222); Fang et al. (216); Ram et al. (223); Guo et al. (224)
	
	Se	Research	Fang et al. (216); Chen et al. (225); Ros et al. (226); Premarathna et al. (227); Xu and Hu (228); Giacosa et al. (229); Liu and Gu (230)

**Wheat**			

	Iron	Research	Aciksoz et al. (231)
	
	Zinc	Research	Cakmak et al. (232); Yang et al. (233)
	
	Se	Research	Aro et al. (207)
	
	P fertilizer + mycorrhiza	Research	Noori et al. (234)
	
	Organic + chemical fertilizers (iron)	Research	Ramzani et al. (235)
	
	*Bacillus aryabhattai* (zinc)	Research	Ramesh et al. (236)

**Maize**			

	Zinc	Research	Alvarez and Rico (237); Lopez-Valdivia et al. (238); Fahad et al. (239); Wang et al. (240); Zhang et al. (241)
	
	Se	Research	Ros et al. (226)
	
	Plant growth-promoting rhizobacteria + Cyanobacteria (zinc)	Research	Prasanna et al. (242)

**Barley**			

	Biofertlizers + NPK fertilizers + Vermicompost	Research	Farahani et al. (243)

**Sorghum**			

	Mycorrhiza + Bacteria	Research	Dhawi et al. (244); Dhawi et al. (245)
	
	Farmyard manure + biofertilizer	Research	Patidar and Mali (246)
**LEGUMES/PULSES**			
**Soybean**			

	Se	Research	Yang et al. (247)

**Chickpea**			

	Actinobacteria (iron, zinc, calcium, copper, manganese, Mg)	Research	Sathya et al. (248)
	
	Plant Biomass, iron, zinc through mycorrhizal inoculation	Research	Pellegrino and Bedini (249)
	
	Zinc	Research	Shivay et al. (250)
	
	Se	Research	Poblaciones et al. (251)

**Pea**			

	Zinc	Research	Poblaciones and Rengel (252)

**Common bean**			

	Zinc	Research	Ibrahim and Ramadan (253); Ram et al. (223)
	
	N, P, K, copper, manganese, zinc (organic + chemical fertilizers)	Research	Westermann et al. (254)
**OILSEED**			
**Canola**			

	Protein, oleic acid, linoleic acid	Research	Nosheen et al. (52)

**Mustard**			

	*Se*, rhizosphere bacteria	Research	Yasin et al. (255)
**VEGETABLES**			
**Potato**			

	Zinc	Research	White et al. (198)
	
	Se	Research	Poggi et al. (256); Cuderman et al. (257)

**Sweet potato**			

	Beta-carotene	Research	Laurie et al. (258)

**Carrot**			

	Iodine, Se	Research	Smolen et al. (259)

**Lettuce**			

	Iodine, Se	Research	Smolen et al. (260)
	
	Se	Research	Carvalho et al. (261)
**FRUIT**			
**Tomato**			

	Iodine	Research	Landini et al. (262)

*Physical application of nutrients, growth-promoting soil microorganisms, N_2_-fixing bacteria and mycorrhizal fungi are utilized to increase the mineral concentration in edible produce*.

## Cereals

### Rice Agronomic Biofortification

Micronutrient biofortification through agronomical practices is an alternative strategy to reduce the iron and zinc deficiency in rice grain. Biofortification of rice plants by foliar spray of iron was an effective way to promote iron concentration in rice grains (214–216). Similarly, fortifying germinating rice plantlets with ferrous sulfate lead to increase iron concentration in germinated brown rice [up to 15.6 times the control (215)]. Foliar application of zinc has been reported as an effective agronomic practice to promote rice grain zinc concentration and zinc bioavailability (216, 218–223). On the other hand, application of zinc to soil as fertilizer in addition to a foliar spray proves to be an important strategy to increase the grain zinc content of rice grown in soils with low background levels of zinc (224). Selenium, which is an essential trace element for human health and proved to be a potent antioxidant, has been also increased by the application of selenate as a foliar spray or as fertilizer in rice (216, 225–230).

### Wheat Agronomic Biofortification

Agronomic biofortification has been very efficiently utilized in wheat grain quality improvement. Inclusion of iron in foliar urea fertilizers has been positively correlated with high iron accumulation (231). Application of foliar zinc has reduced human zinc deficiency in regions with potentially zinc-deficient soil and also improved its bioavailability by reducing antinutrient factors like phytic acid (233). Due to significant effects of zinc fertilizers on grain yield, the total amount of zinc-containing NPK fertilizers increased from 0 in 1994 to a record level of 400,000 t per annum in 10–15 years in Turkey. Use of zinc-containing fertilizers increased zinc concentration in grain, and obviously contributed to human nutrition and health in Turkey, especially in rural areas, where wheat provided more than 50% of the daily calorie intake (206). Agronomic biofortification of Se in wheat has been adopted with success in Finland (207). Compound fertilizers supplemented with Se were utilized since 1984, and it resulted in an increase in human serum selenium. Apart from chemical and organic fertilizers, researchers have also investigated the role of biofertilizers in promoting the yield of grains. Mycorrhizal fungi along with fertilizers are extensively being used for biofortification (234). Iron biofortification of wheat grains has been accomplished through integrated use of organic and chemical fertilizers and zinc biofortification by using *Bacillus aryabhattai* (235, 236).

### Maize Agronomic Biofortification

Among micronutrients, zinc is required for obtaining nutrient-enriched grain and optimum yield in maize. For achieving this, various zinc fertilizer treatments and foliar applications have been carried out in maize crop (237, 239–241). Plant growth-promoting rhizobacteria have led to nutrient enrichment in the plants and have been included in agronomic approaches to develop effective biofortification strategies for the staple crops. One of the effective examples is the maize crop with increased zinc content (242). The Selenium (Se) importance in human and animal health has been known worldwide, and it has also been increased by applying fertilization as an effective agronomic biofortification strategy (226).

### Barley Agronomic Biofortification

The micronutrient profile of barley has been improved by the application of various organic and inorganic biofertilizers. The concentration of zinc and iron in grains has been enhanced by the application of biofertilizers along with inorganic fertilizers and vermicompost (243).

### Sorghum Agronomic Biofortification

Sorghum is cultivated worldwide for grain and fodder. This crop often suffers from the challenge of growing in nutrient poor and contaminated soil. Its nutrient profile has been promoted by the application of fertilizers (both organic and inorganic) that have an additive effect on the yield. Researchers have intended to improve the nutrient uptake and alter the metabolic profile of sorghum by using the combination of plant growth-promoting bacteria and arbuscular mycorrhizal fungi (AMF) (244, 245). Also, the inoculation of *Azospirillum* alone and in combination with phosphate-solubilizing bacteria increased sorghum grain yield and protein content by improving the status of phosphorous and nitrogen in the soil (246).

## Legumes

### Soybean Agronomic Biofortification

Selenium-enriched soybean has been produced by the foliar application of selenium complex salts as fertilizers (247).

### Chickpea Agronomic Biofortification

Chickpea has been targeted for the mineral deficiencies, especially the mineral iron, zinc, calcium, copper, manganese, and Mg by using plant growth-promoting actinobacteria (248). Chickpea biofortification for iron and zinc has been addressed by using AMF (249). Similarly, zinc and Se have been fortified in chickpea by foliar spray of respective minerals (250, 251).

### Pea Agronomic Biofortification

Field peas are the second largest legume crop worldwide, also known for their high protein content and its enrichment for zinc has been obtained with foliar zinc applications alone or in combination with soil zinc applications (252).

### Common Bean Agronomic Biofortification

A common bean is an herbaceous annual plant grown for edible dry grain. Beans are a good vehicle for zinc biofortification and have been enriched with zinc by the application of foliar zinc fertilizer (223, 253). Furthermore, it has been studied that administration of organic and chemical fertilizers stimulated the uptake of N, P, K, copper, manganese, and zinc in common bean (254).

## Oilseeds

### Canola Agronomic Biofortification

Canola supplemented with plant growth-promoting rhizobacteria *viz*. *Azospirillum brasilense, Azotobacter vinelandii* along with chemical fertilizers resulted in increased protein, oleic acid, and linoleic acid content in the seed which indicated that rhizobacteria are highly effective in improving yield and nutritive value of canola oil (263).

### Mustard Agronomic Biofortification

Mustard has been targeted for Se enhancement. Plant uptake of Se as selenate has been enhanced by rhizosphere bacteria from a seleniferous area (255).

## Vegetables

### Potato Agronomic Biofortification

Field experiments were undertaken to increase zinc concentrations in potato tubers (both flesh and skin of tubers) using foliar zinc fertilizers, which significantly increased tuber zinc concentrations. It was also found that zinc oxide and zinc sulfate were more effective than zinc nitrate as foliar fertilizers for increasing tuber zinc concentrations while maintaining yields (264). Increase in Se content of potato tubers has been reported after foliar application of selenium, selenite, and selenate to potato (256, 257). Foliar application of selenium with humic acids was proven to be a good way to increase the selenium content of potatoes (256).

### Sweet Potato Agronomic Biofortification

Increase in beta-carotene in orange-fleshed sweet potato has been observed with irrigation and chemical fertilizer treatments (258).

### Carrot Agronomic Biofortification

Carrot leaves and storage roots have been supplemented with I and Se by application of both as fertilizers. It has been reported that consumption of 100 g fresh weight of carrots fertilized with I and Se (KICNa_2_SeO_3_, KIO_3_CNa_2_SeO_3_) can supply 100% of the recommended daily allowance (259).

### Lettuce Agronomic Biofortification

Lettuce I and Se biofortification have been achieved by the application of KIO_3_ and Na_2_SeO_4_ as foliar spray and nutrient medium (260). Lettuce Se biofortification in the leaves has been carried out with good results after soil agronomic biofortification with an inorganic form of selenium (261).

## Fruit

### Tomato Agronomic Biofortification

Studies have concluded that a tomato is an excellent crop for iodine biofortification programs when treated with iron fertilizers (262).

### Biofortification through Conventional Breeding—Most Trusted Approach

Biofortification through conventional breeding in the most accepted method of biofortification. It offers a sustainable, cost-effective alternative to transgenic- and agronomic-based strategies. Sufficient genotypic variation in the trait of interest is necessary for conventional breeding to be feasible. Breeding programs can utilize this variation to improve the levels of minerals and vitamins in crops. In conventional plant breeding, parent lines with high nutrients are crossed with recipient line with desirable agronomic traits over several generations to produce plants with desired nutrient and agronomic traits. However, breeding strategies have to sometimes rely on the limited genetic variation present in the gene pool. In some cases, this can be overcome by crossing to distant relatives and thus moving the trait slowly into the commercial cultivars. Alternatively, new traits can be introduced directly into commercial varieties by mutagenesis.

Because this approach is likely to be the most expedient method to improve plants, several international organizations have initiated programs to improve the nutritional content of crops through breeding programs. The Health grain Project (2005–2010) involving 44 partners from 15 countries and over £10 million was carried out in the European Union to develop health promoting and safe cereal foods and ingredients of high eating quality. It has since developed into the Healthgrain forum with a wide range of participants from academia and industry. More than 100 publications have reported bioactive compounds in whole-grain cereals, genetic variation, heritability, and effect on reducing risks of many lifestyle-related diseases (265–267). The CGIAR along with the International Center for Tropical Agriculture (CIAT) and the International Food Policy Research Institute have launched the HarvestPlus program to breed biofortified staple food crops. HarvestPlus is investing heavily to boost three key nutrients-vitamin A, iron, and zinc and is targeting the staple crops, wheat, rice, maize, cassava, pearl millet, beans, and sweet potato in Asia and Africa (268). It is directed to produce staple food crops with enhanced levels of bioavailable essential minerals and vitamins that will have measurable impact on improving the micronutrient status of target populations, primarily resource-poor people in the developing world. The Biocassava Plus program had been initiated to improve the nutrition status of cassava crop. Due to better acceptability, large numbers of crops have been targeted for biofortification through crop breeding (Table 4).

**Table 4:** Tabulation of crops, nutrients, research status, and concerned publications on biofortification through breeding.

Type of cereal	Type of biofortification	Status	Variety/country	Paper/Source
**CEREALS**				
**Rice**				

	Zinc Iron	**Released**	**Bangladesh**: BRRIdhan 62, BRRIdhan 72, BRRIdhan 64	CIAT, HarvestPlus
	
	Iron	Research Traditional variety/Research	India, Philippines: IR68144-3B-2-2-3 (improved line) Jalmagna	IRRI Gregorio et al. (269)
	
	Zinc	Traditional variety/Research	Jalmagna	Gregorio et al. (269)

**Wheat**				

	Zinc	**Released**	**India**: BHU 1, BHU 3, BHU 5, BHU 6, BHU 17, BHU 18 **Pakistan**: NR 419, 42, 421, Zincol	CIAT, CIMMYT, HarvestPlus
	
	Zinc and iron	**Released**	**India**: WB2	Indian Institute of Wheat and Barley Research, India
	
	Zinc	**Released**	**India**: PBW1Zn	Punjab Agricultural University, India
	
	Zinc and iron	Research		Cakmak et al. (208); Monasterio and Graham (270); Welch et al. (271); Cakmak et al. (272)
	
	Carotene	**Released**	**India**: HI 8627	IARI
	
	Lutein	Research		Digesu et al. (273); Ficco et al. (274)
	
	Anthocyanins (colored wheat)	**Released**	**China**: Black-grained wheat	Havrlentova et al. (276)
	
		**Released**	**Austria**: Indigo	Havrlentova et al. (276)
	
		Registered	**Slovakia**: PS Karkulka	Havrlentova et al. (276)
	
		Registered/Research	**India**: NABIMG-9, NABIMG-10, NABIMG-11	Garg et al. (275)
	
		Research		Havrlentová et al. (276); Martinek et al. (277)

**Maize**				

Orange Maize	Vitamin A	**Released**	**Zambia**: GV662A, GV664A, GV665A **Nigeria**: Ife maizehyb-3, Ife maizehyb-4, Sammaz 38 (OPV), Sammaz 39 (OPV) **Ghana**: CSIR-CRI Honampa (OPV)	CIMMYT, International Institute of Tropical Agriculture (IITA), HarvestPlus

Quality Protein Maize	Lysine and Tryptophan	**Released**	**India**: CML176, CML176 × CML186, HQPM-1, HQPM4, HQPM-5, HQPM-7, VivekQPM-9, FQH-4567 **China**: CML140, CML194, P70 **Vietnam**: CML161 × CML165 **Mexico**: CML142 × CML176, CML142 × CML150, CML176, CML170, CML186 × CML149, CML176 × CML186 **South Africa**: QS-7705 **Ghana**:GH-132-28 **Guinea**: Obatampa **Benin**: Obatampa **Uganda**: Obangaina **Mozambique**:Susuma **Brazil**: BR-451, BR-473 **Venezuela**: FONAIAP **Peru**: INIA **Colombia**: ICA **Honduras**: HQ-31 **El Salvador**: HQ-61 **Guatemala**: HB-Proticta **Nicaragua**: NB-Nutrinta, HQ INTA-993	Surinder Vasal and Evangelina Villegas, CIMMYT
	
	Provitamin A carotenoidsTotal carotenoids	Research		Palmer et al. (278)
	
	Carotenoids, vitamin E and phenolic compounds	Research		Muzhingi et al. (279)
	
	Anthocyanins	Research		Lago et al. (280)
	
	Fatty acids + vitamin E	Research		Goffman and Böhme (281)

**Sorghum**				

	Iron	**Released**	**India**: ICSR 14001, ICSH 14002 Hybrids: ICSA 661 × ICSR 196, ICSA 318 × ICSR 94, ICSA 336 × IS 3760	ICRISAT, HarvestPlus
	
	Iron	**Released**	**Nigeria**: 12KNICSV (Deko)-188 12KNICSV-22 (Zabuwa)	ICRISAT, HarvestPlus
	
	Iron, zinc, beta-carotene	Research		Reddy et al. (282)

**Millets**				

	Iron and zinc(Pearl Millet)	**Released**	**India**: Dhanashakti Hybrid ICMH 1201 (Shakti-1201)	ICRISAT, HarvestPlus
	
	Iron and zinc	Research		Velu et al. (283); Rai et al. (284)
**LEGUMES/PULSES**				
**Lentils**				

	Iron and zinc	**Released**	**Bangladesh**: Barimasur-4, Barimasur-5, Barimasur-6, Barimasur-7, Barimasur-8 **Nepal**: ILL 7723-Khajurah-1, Khajurah-2, Shital, Sisir, Shekhar and Simal **India**: L4704 and Pusa Vaibhav **Ethiopia**: Alemaya **Syria**: Idlib-2 and Idlib-3	ICARDA, HarvestPlus

**Cow Pea**				

	Iron	**Released**	**India**: Pant Lobia-1, Pant Lobia-2, Pant Lobia-3, Pant Lobia-4	G.B. Pant Agriculture University, HarvestPlus

**Beans**				

	High iron and zinc	**Released**	**Rwanda**: RWR 2245; RWR 2154; MAC 42; MAC 44; CAB 2; RWV 1129; RWV 3006; RWV 3316; RWV 3317; RWV 2887	HarvestPlus (Rwanda)
	
	Iron	Research		Blair et al. (285); Gelin et al. (286); Beebe et al. (287)
	
	Zinc	Research		Blair et al. (285); Gelin et al. (286); Beebe et al. (287)
**VEGETABLES**				
**Potato**				

	Antioxidants	Research		Lachman, et al. (288); Andre et al. (289)
	
	Zinc, iron	Research		Burgos et al. (290); Brown et al. (291)
	
	Copper, iron, manganese and zinc	Research		Haynes et al. (292)

**Sweet potato**				

Orange Sweet Potato	Vitamin A	**Released**	**Uganda**: Ejumula, Kakamega, Vita, Kabode, Naspot 12O, Naspot 13O **Zambia**: Olympia, Twatasha, Kokota, Chiwoko, Zambezi	HarvestPlus, International Potato Centre (CIP)

	Beta-amylase	Research		Kumagai et al. (293)

**Cauliflower**				

	Beta-carotene	**Released**	**India**: Pusa Betakesari **New York**: Purple Graffiti, Orange Cheddar	IARI, IndiaCornell University, New York

**Cassava**				

	Vitamin A	**Released**	**Nigeria**: TMS 01/1368—UMUCASS 36, TMS 01/1412—UMUCASS 37, and TMS 01/1371—UMUCASS 38, NR 07/0220—UMUCASS 44, TMS 07/0593—UMUCASS 45 and TMS 07/539—UMUCASS 46 **DRC**: Kindisa (TMS 2001/1661)	IITA, HarvestPlus
	
	Iron	Research		Maziya-Dixon et al. (294); Chavez et al. (295)
	
	Carotenes	Research		Maziya-Dixon et al. (294); Chavez et al. (295)
**FRUITS**				
**Tomato**				

	Anthocyanin	Research	Italy: Sun Black Israel: Black Galaxy	Mazzucato et al. (296)

**Banana**				

	Vitamin A	**Released**	**DRC and Burundi**: Apantu, Bira, Pelipita, Lai, To’o	Bioversity International—Uganda, HarvestPlus

**Mango**				

	Beta-carotene	**Released**	**India**: Amarpali, Pusa Arunima, Pusa Surya, Pusa Pratibha, Pusa	IARI, India
	Vitamin C		Peetamber, Pusa Lalima, and Pusa Shreshth	
	Beta-carotene	Research	Mexico: Ataulfo	
	Vitamin C			USDA Agricultural Research Service

**Grapes**				

	Antioxidants	**Released**	**India**: Pusa Navrang	IARI, India

*Breeding is so far the best method for crop biofortification. Large number of biofortified cultivars have been released by this approach that are helping in addressing the challenge of micronutrient malnutrition prevalent in the developing countries*.

*Released varieties and their country of release have been bold faced*.

## Cereals

### Rice Breeding

Rice is greatly emphasized for micronutrient enhancement. It is one of the most consumed staple food crop and its biofortification can have a significant effect on malnutrition challenge. The milled rice is poor source of minerals. Different old rice varieties with high iron and zinc content in grain have been screened and the higher mineral trait has been combined with improved agronomic traits by breeding methods. The world’s first zinc enriched rice varieties developed by HarvestPlus were released in 2013 by the Bangladesh Rice Research Institute (BRRIdhan 62, BRRIdhan 72, and BRRIdhan 64), which is claimed to contain 20–22 ppm zinc in brown rice. In India and Philippines, an improved line (IR68144-3B-2-2-3) was identified in a cross between a high-yielding variety (IR72) and a tall, traditional variety (Zawa Bonday) with a high concentration of grain iron [about 21 ppm in brown rice (269)]. Similarly, Jalmagna, a traditional variety which had almost double the iron concentration of common rice variety and zinc concentration, nearly 40% more than that of common rice variety has been identified for further breeding programs to improve iron and zinc concentration (269).

### Wheat Breeding

Wheat as a staple crop is the first and foremost target for biofortification. Wide variation in grain iron and zinc concentrations in wheat and its closely related wild species has been observed that it can be exploited for improvement of modern elite cultivars (270, 272, 297). Utilizing this variation HarvestPlus has released several varieties of wheat with 4–10 ppm higher zinc content. Six varieties of high zinc wheat (BHU 1, BHU 3, BHU 5, BHU 6, BHU 7, and BHU 18) were released in India in 2014 followed by the release of four varieties in Pakistan in 2015 (NR 419, 42, 421, and Zincol). Two varieties BHU 1 and BHU 6 have high yield, disease resistance in addition to high zinc. Recently, variety with high zinc (PBW1Zn) has been released by Punjab Agricultural University, India. Another variety with high zinc and iron content (WB2) has been developed and released by Indian Institute of Wheat and Barley Research, India. Apart from releasing cultivars, several researchers have reported an increase in the zinc and iron content of wheat by plant breeding (208, 270–272). Provitamin A has been another important nutrient targeted for biofortification through breeding. High provitamin A durum wheat variety (HI 8627) has been released by the Indian Agricultural Research Institute (IARI), India in 2005. Several new cultivars have been released after that with the improved beta-carotene content. Yellow pigment content (YPC; carotenoids mainly xanthophyll lutein) in durum wheat is an important quality trait and an antioxidant. A large number of recent durum wheat varieties released in different countries in the past decade show significantly higher YPC than the old varieties released before the 1970s [(273, 274) and others]. Improvement of antioxidant properties contributed by anthocyanins had also been an area of significant research in wheat. Colored wheat (black, blue, and purple) trait has been used in several breeding programs in different countries. Black-grained wheat cultivar has been released in China after more than 20 years running effort in breeding and has been reported to be high in protein content and selenium (298). The purple wheat cultivar Indigo has been released in Austria in 2006 (299). The purple wheat cultivar PS Karkulka has been registered in Slovakia in 2014. Purple, blue, and black white lines have been developed and registered in India in 2017 (275). The importance of colored wheat can be adjudged from the patent on functional foods from colored wheat in China (CN102217664 B). Apart from this several researchers have worked on different aspects of colored wheat [reviewed in Ref. (276, 277)].

### Maize Breeding

Maize is a cash crop grown for animal feed, industrial purposes (source of sugar, oil, starch, and ethanol) and for use for human consumption. The vast genetic diversity of maize has been the basis for the breeding programs that have generated much of the higher yielding maize used worldwide. Scientists have discovered varieties that have naturally high levels of provitamin A. HarvestPlus is using these lines to breed high-yielding varieties of biofortified maize with higher levels of provitamin A to combat vitamin A deficiency. The provitamin A maize is one of the significant achievements in the field of biofortification. Biofortified orange maize varieties have been grown commercially in Zambia (GV662A, GV664A, and GV665A), Nigeria {Ife maizehyb-3, Ife maizehyb-4, Sammaz 38 (OPV), Sammaz 39 (OPV)} and Ghana {CSIR-CRI Honampa (OPV)} since 2013 (300). Malawi, Zimbabwe (ZS242) and Tanzania have also released biofortified orange maize recently (301). As a positive effect an increase in pupillary response was observed among Zambian children consuming vitamin A biofortified maize (301). Breeders have evaluated antioxidants like tocochromanols, oryzanol, and phenolic compounds in proVA biofortified maize (279). Another significant achievement in the field of maize biofortification is quality protein maize (QPM). Maize breeders have developed QPM with high essential amino acids lysine and tryptophan by incorporating opaque-2 (o2) mutant gene from naturally occurring maize into the maize cultivars. International Maize and Wheat Improvement Center (CIMMYT) has released such hybrid varieties in India (CML176, CML176 × CML186, HQPM4, HQPM-7, VivekQPM-9, HQPM-5, HQPM-1, FQH-4567), China (CML140, CML194, P70), Vietnam (CML161 × CML165), Mexico (CML142 × CML176, CML142 × CML150, CML176, CML170, CML186 × CML149, CML176 × CML186), South Africa (QS-7705), Ghana (GH-132-28), Guinea (Obatampa), Uganda (Obangaina), Benin (Obangaina), Mozambique (Susuma), Brazil (BR-451, BR-473), Venezuela (FONAIAP), Peru (INIA), Colombia (ICA), Honduras (HQ-31), El Salvador (HQ-61), Guatemala (HB-Proticta), and Nicaragua (NB-Nutrinta, HQ INTA-993). For QPM maize breeders, Surinder Vasal and Evangelina Villegas won 2000 world food prize. Maize has also been inbred by recurrent selection scheme, to increase the carotenoids (278) alone or in combination of vitamin E and phenolics (279) and antioxidant power (280). Attempts have been made to increase its vitamin E content (281).

### Sorghum Breeding

The prospects of breeding for micronutrients and beta-carotene rich sorghums have been discussed by Reddy et al. (282). Sorghum varieties have been screened for high minerals, protein (302), lutein, zeaxanthin, and beta-carotene contents (303). Sorghum germplasm has shown large variability and genetic heritability for iron and zinc content (304). Biofortified iron rich sorghum lines (ICSR 14001, ICSH 14002) and hybrids (ICSA 661 × ICSR 196, ICSA 318 × ICSR 94, ICSA 336 × IS 3760) have been bred by ICRISAT and released in India.

New nutritionally high (Fe) sorghum varieties (12KNICSV-22 and 12KNICSV-188) have been released in Nigeria that may boost the malnourished populations, especially children in Nigeria. One of the new varieties (12KNICSV-188) has iron content three times higher than typically grown sorghum. These new varieties involved crossing local Nigerian germplasm with improved lines from ICRISAT (Mali).

### Millets Breeding

Pearl millet is the cheapest source of iron and zinc (305) and large variation has been seen in its germplasm for these micronutrients (283). In India, biofortified (iron and zinc) pearl millet variety “Dhanashakti” and a hybrid ICMH 1201 (Shakti-1201) has been released by ICRISAT, HarvestPlus in 2014. Besides that, two varieties, ICMH 1202 (Nirmal-7) and ICMH 1301, are currently undergoing advanced farm trials. Various well-adapted commercial varieties, their progenies, and hybrids containing high content of iron and zinc in grain have been reported (283, 284).

## Legumes and Pulses

### Lentil Breeding

Lentil, a key pulse in many dryland countries and has easy to cook properties. It has been directed by ICARDA, HarvestPlus for biofortification of iron and zinc with the help of breeding process using genetic diversity stored in gene banks. Research findings have shown that there is a positive correlation of iron and zinc synthesis with protein synthesis, therefore lentil varieties with higher iron, zinc, and protein content can be developed together [ICARDA, HarvestPlus (306)]. High iron and zinc lentil varieties, five in Bangladesh (Barimasur-4, Barimasur-5, Barimasur-6, Barimasur-7, and Barimasur-8), seven in Nepal (ILL 7723, Khajurah-1, Khajurah-2, Shital, Sisir Shekhar, Simal), two in India (L4704, Pusa Vaibhav), one in Ethiopia (Alemaya), and two in Syria (Idlib-2, Idlib-3) has been released by ICARDA, HarvestPlus biofortification program till date. Lentil varieties have been screened for variation in Se content (307).

### Cow Pea Breeding

Cow pea which is also known as poor man meat, rich in protein content has been biofortified for iron content by means of breeding methods. Pant Lobia-1 (2008), Pant Lobia-2 (2010), Pant Lobia-3 (2013), and Pant Lobia-4 (2014) varieties with increased iron content have been released by GB Pant University, Pantnagar, India in collaboration to HarvestPlus.

### Bean Breeding

Studies till date suggest that the iron content of the common bean (*P. vulgaris*) could be increased by 60–80%, while zinc content would be more modest, perhaps around 50%. High heritability has been observed in iron and zinc content in common bean (285, 287, 308). Genes associated with zinc accumulation have been identified in navy bean (286). HarvestPlus is working in this direction and promoting iron biofortified beans in several developing countries. They have released 10 Fe-biofortified common bean varieties in Rwanda (RWR 2245, RWR 2154, MAC 42, MAC 44, CAB 2, RWV 1129, RWV 3006, RWV 3316, RWV 3317, and RWV 2887). HarvestPlus also released ten biofortified iron bean varieties in the Democratic Republic of Congo, i.e., COD MLB 001, COD MLB 032, HM 21-7, RWR 2245, PVA 1438, COD MLV 059, VCB 81013, Nain de Kyondo, Cuarentino, Namulenga.

## Vegetables

### Potato Breeding

Potato tubers are the richest sources of antioxidants in human diet. The natural variation of cultivated potato germplasm containing red and purple pigment could possibly represent the contribution of the potatoes to the portion of antioxidants in human nutrition. Therefore, effort of breeders focuses on the breeding of such variants (288). Furthermore, vast genetic variation for micronutrients (291) exists in potato that can be exploited for breeding to further increase iron and zinc levels in human diets (290). A genetically diverse sample of potato cultivars native to the Andes of South America has been obtained from a collection of nearly 1,000 genotypes and evaluated as a source of antioxidants and minerals (copper, iron, manganese, and zinc) (289, 292). International potato center (CIP) and HarvestPlus have developed high iron and zinc advanced breeding material after crossing diploid Andean landrace potatoes with high zinc and iron with disease resistant tetraploid clones. The main target countries for biofortified potato are Rwanda and Ethiopia. National Institute for Agrarian Innovation’s (INIA) Potato Program has developed the INIA 321 Kawsay variety in Peru that has a high content of iron and zinc.

### Sweet Potato Breeding

Developing countries are growing 95% of the world’s sweet potato crop, where malnutrition is the biggest problem. The sweet potato has been targeted for improvement in vitamin A. HarvestPlus and International Potato Centre (CIP) have developed and released several varieties of orange sweet potato with high vitamin A. Six varieties have been released in Uganda (Ejumula, Kakamega, Vita, Kabode, Naspot 12O, and Naspot 13O) and three in Zambia (Twatasha, Kokota, and Chiwoko). Zambia Agriculture Research Institute has successfully completed the development of 15 new varieties of vitamin A fortified sweet potatoes. The HarvestPlus orange sweet potato consumption had a significant effect on household food and nutritional security in Sub Saharan Africa, and for this contribution; they have been recently honored with World Food Prize-2016. Furthermore, researchers have identified several sweet potato genotypes that completely lack or have only traces of β-amylase in their storage roots. Such verities could facilitate the breeding of sweet potato for low β-amylase content which can be potentially used for processing and as a staple food (293).

### Cauliflower Breeding

*Brassica oleracea* including cauliflower gene pool has been screened for genetic variation of zinc concentration and sufficient natural variation has been identified (309). The provitamin A (beta-carotene) rich orange colored cauliflower variety (Pusa BetaKesari; 800–1,000 μg/100g) has been released by the Indian Agricultural Research Institute (IARI). Now numbers of colored cauliflower verities are known at world level, having orange and purple color rich in beta-carotene and anthocyanin, respectively. Colored cauliflower varieties, Purple Graffiti and Orange Cheddar, have been developed by Cornell University, USA.

### Cassava Breeding

Cassava is a staple vegetable root crop in developing countries, especially in Africa, Latin America, and the Caribbean. In the African continent, it has been targeted for alleviation in provitamin A (beta-carotene) by HarvestPlus in collaboration with International Institute of Tropical Agriculture. Under these collaborations, they have released six vitamin A fortified varieties in Nigeria (2011; TMS 01/1368—UMUCASS 36, TMS 01/1412—UMUCASS 37 and 2014; TMS 01/1371—UMUCASS 38 and NR 07/0220—UMUCASS 44, TMS 07/0593—UMUCASS 45, and TMS 07/539—UMUCASS 46) and one in DRC-Democratic Republic of Congo [Kindisa (TMS 2001/1661)]. Cassava also has a wide range of genotype differences for total carotene, proteins, and minerals (iron and zinc) which has led to the development of improved nutritive value cassava crop (294, 295).

## Fruits

### Tomato Breeding

Tomato is a highly valuable crop and an important source of vitamin A and C. Genetically diverse wild population of tomato has been investigated intensively for specific traits and exploited in tomato breeding (310). Anthocyanin biofortified tomato “Sun Black” with deep purple fruit pigmentation due to high anthocyanin content in the peel has been developed by conventional breeding approach (296). Another variety “Black Galaxy” generated by similar approach has been reported from Israel.

### Banana Breeding

Breeding banana is difficult and expensive, as commercial varieties are sterile triploids (3×) and also a high degree of cross incompatibility can exist among the fertile groups. For combating this problem, large scale screening of several banana germplasm for the identification of high levels of provitamin A has been carried out in the Democratic Republic of Congo (DRC) and Burundi by Biodiversity International (BI) in collaboration with HarvestPlus. In this program, they released five varieties (Apantu, Bira, Pelipita, Lai, and To’o) rich in provitamin A in Eastern DRC and Burundi.

### Mango Breeding

Mango offers a natural source of beta-carotene, vitamin C, and valuable antioxidants but their nutrient levels vary with mango variety. It has been observed that most of the mango varieties provide more than recommended daily value of vitamin C and beta-carotene. Mango also contains a variety of phenolics like ellagic acid, gallotannin, and mangiferin (311). The Mexican-grown Ataulfo variety ranked highest in both vitamin C (ascorbic acid) and beta-carotene (USDA’s Agricultural Research Service). In India, IARI introduced many varieties with enhanced nutritional and agronomical important characters.

### Grape Breeding

Grapes have high mineral content, including high vitamins C and K, and are a natural source of antioxidants and other polyphenols, and offer a variety of additional health benefits. Phenolic compounds and antioxidant properties of different grape cultivars grown in China have been assessed (312). The Indian Agricultural Institute has released an improved variety, i.e., Pusa Navrang which contains higher amount of total soluble solids (carbohydrates, organic acids, proteins, fats, and minerals) and antioxidants.

## Limitations of Biofortification

### Limitations in Agronomic Biofortification

Application of fertilizers fortified with micronutrients is the simplest method among all biofortification methods. But the success of agronomical biofortification is highly variable due to the differences in mineral mobility, mineral accumulation among plant species, soil compositions in the specific geographical location of each crop (313). For example, a study involving diverse rice genotypes indicated that, in the phosphate deficient soils due to reduction in the root biomass, differences in the phosphate uptake among the genotypes were as high as 20-fold (314). Soil composition analysis has indicated that almost 1/2 of the agricultural soils of India, 1/3 of China, 14 Mha of Turkey, 8 Mha of Australia are zinc deficient (315). Agronomic biofortification is less cost-effective and labor intensive as it demands continuous inputs, through the application of micronutrient to the soil or plant regularly. Furthermore, it is not always possible to target the micronutrient into edible plant parts like seed or fruit and can sometimes result in the accumulation of desired nutrients in the leaves or other non-edible portions of plants; therefore, this technique is only successful in certain minerals and specific plant species. For instance, higher zinc efficiency in cereals grown in zinc deficient soils in Turkey was associated with higher uptake of zinc from the soil, but not with increased accumulation of zinc in the grain (208). Furthermore, mineral bioavailability hindered by antinutrient compound like phytic acid is another major challenge (316). In addition, the biggest of all constraints is that the fertilizers accumulation in soil and water poses adverse environmental effects (317).

### Limitations in Conventional Breeding Methods

The design of conventional plant breeding programs to improve micronutrient content has proved to be successful and is a sustainable and cost-effective solution in the long run; however, there are limitations with respect to the amount of genetic variability for the micronutrients in the plant gene pool and the time needed to generate cultivars with the desired trait(s). In some cases, this can be overcome by crossing to distant relatives and thus introgressing traits into commercial cultivars, but in many occasions, it would be impossible to breed for a specific trait using conventional means, and the timescale and effort involved may be quite unrealistic, e.g., improving Se concentration in wheat grains (318) and improvement of oleic, linoleic, and linolenic fatty acid content in soybean (319). In general, improvement in oil quality has been targeted with better results with transgenic-based approach (Figure 3B) due to limited variability, heritability, and linkage drag.

### Limitations in Transgenic Methods

Transgenic crops overcome the limitation of restricted genetic variation among plants as in the case of conventional breeding but the major limitation of this method is its low acceptance among masses. It is very important that the biofortified crops be readily adapted by farmers and community in significant enough numbers to improve the general nutritional health of a given community (320). Another limitation is that different countries have adopted different regulatory processes for the acceptance and commercialization of these transgenic crops. Regrettably, the current political and economic landscape is not receptive to this technology (321). Furthermore, these regulatory processes are very expensive and time consuming (322). Let us take the example of Bt Brinjal. It has been initially developed by Mahyco, an Indian seed company. Unfortunately, it was not released in Indian because some of the scientists, farmers, and anti-GMO activists, raised concerns and a moratorium on its release was imposed, until further tests were conducted. However, four varieties of Bt Brinjal were given approval for commercial release in Bangladesh in 2013–2014. Although the research efforts devoted to the transgenic-based approach are quite higher compared with breeding based, its success rate in terms of cultivar release in very low (Figure 3A) due to time required from target trait and gene identification, modification, expression, and assessment of agronomical traits to understanding the possible effect on other life forms. For example, after 8 years project, the scientific details of the Golden rice were first published in Science in 2000 (41), and since then different groups, including International Rice Research Institute scientists are working on it, but Golden Rice is still not ready for farmers due to issues with its yield. Its dissemination is also being held back due to inability to get approval from Governments.

### Other Limitations

The postharvest processing of each crop must be considered to optimize biofortification strategies. For example, the seeds of many cereals are often consumed after milling or polishing. Although the concentrations of some essential mineral elements, such as Se and S, are highest in the embryo, others, such as iron, zinc, and copper, are highest in the bran (269, 317). Milling or polishing cereal seeds can, therefore, remove large quantities of minerals from the diet; the extent of these losses is genotype dependent (269). In addition, the presence of certain antinutrients in crops reduces the bioavailability of certain nutrients in crops. For examples, antinutrients like phytate, tannins, oxalate, fiber, and hemaglutinins reduce the bioavailability of minerals in human gut (20, 101). Furthermore, in the context of global environmental change, approaches for improving food production, improvements in a crop’s ability to maintain yields with lower water supply and quality will be critical. In addition, numerous genes are involved in controlling the amount of a mineral element that is absorbed by roots, translocated to shoot, remobilized from vegetative tissues, and deposited in edible portions of seeds and grains in forms that are utilizable in persons consuming the crop (323, 324). Considerations must also include the micronutrient concentrations in the edible portions of crops, and the amount of nutrients that can be absorbed by the consumer, after processing and cooking (325).

## Conclusion

It is well established that biofortification is a promising, cost-effective, agricultural strategy for improving the nutritional status of malnourished populations throughout the world. Biofortification strategies based on crop breeding, targeted genetic manipulation, and/or the application of mineral fertilizers hold great potential for addressing mineral malnutrition in humans. The generation of biofortified food crops with improved nutrient contents such as increases in iron, zinc, Se, and provitamin A content are providing sufficient levels of these and other such micronutrients that are frequently lacking in the diets of the developing and developed world. International initiatives, such as the HarvestPlus program and national initiatives, are acting as pillars to achieve these targets. These efforts have delivered crops with the potential to increase both the amounts and bioavailability of essential mineral elements in human diets, especially in staple cereal crops like wheat, maize, cassava, beans, sweet potatoes, and millets. But biofortification of crops is a challenging endeavor. To achieve this, collaboration between plant breeders, nutrition scientists, genetic engineers, and molecular biologists is essential. Traditional breeding approaches are finding widespread and easy acceptance and have been used to enhance the nutritional qualities of foods. Although a greater emphasis is being laid on transgenic means success rates of breeding based approaches are much higher as transgenically fortified crop plants have to face hurdles due to acceptance constraints among consumers and different expensive and time consuming regulatory approval processes, adopted by different countries. Besides these challenges, biofortified crops hold a very bright future as these have the potential to remove micronutrient malnutrition among billions of poor people, especially in the developing countries.

## Author Contributions

MG and NG built the layout of the article, collected literature, and wrote the article. SS and PK collected literature and helped in manuscript writing. AK and VC edited it. PA assisted in reference management.

## Conflict of Interest Statement

The authors declare that the research was conducted in the absence of any commercial or financial relationships that could be construed as a potential conflict of interest.
